# Identification of cardiotoxicity related to non-small cell lung cancer (NSCLC) treatments: A systematic review

**DOI:** 10.3389/fphar.2023.1137983

**Published:** 2023-06-13

**Authors:** Stefanie Ho Yi Chan, Yasmin Khatib, Sherael Webley, Deborah Layton, Sam Salek

**Affiliations:** ^1^ School of Life and Medical Sciences, University of Hertfordshire, Hatfield, United Kingdom; ^2^ IQVIA UK, London, United Kingdom; ^3^ PEPI Consultancy Limited, Southampton, United Kingdom; ^4^ University of Keele, Keele, United Kingdom

**Keywords:** anticancer drugs, cancer treatments, cardiotoxicity, cardiovascular events, non-small cell lung cancer (NSCLC)

## Abstract

**Introduction:** In the last few decades, there has been a rapid development in cancer therapies and improved detection strategies, hence the death rates caused by cancer have decreased. However, it has been reported that cardiovascular disease has become the second leading cause of long-term morbidity and fatality among cancer survivors. Cardiotoxicity from anticancer drugs affects the heart’s function and structure and can occur during any stage of the cancer treatments, which leads to the development of cardiovascular disease.

**Objectives:** To investigate the association between anticancer drugs for non-small cell lung cancer (NSCLC) and cardiotoxicity as to whether: different classes of anticancer drugs demonstrate different cardiotoxicity potentials; different dosages of the same drug in initial treatment affect the degree of cardiotoxicity; and accumulated dosage and/or duration of treatments affect the degree of cardiotoxicity.

**Methods:** This systematic review included studies involving patients over 18 years old with NSCLC and excluded studies in which patients’ treatments involve radiotherapy only. Electronic databases and registers including Cochrane Library, National Cancer Institute (NCI) Database, PubMed, Scopus, Web of Science, ClinicalTrials.gov and the European Union Clinical Trials Register were systematically searched from the earliest available date up until November 2020. A full version protocol of this systematic review (CRD42020191760) had been published on PROSPERO.

**Results:** A total of 1785 records were identified using specific search terms through the databases and registers; 74 eligible studies were included for data extraction. Based on data extracted from the included studies, anticancer drugs for NSCLC that are associated with cardiovascular events include bevacizumab, carboplatin, cisplatin, crizotinib, docetaxel, erlotinib, gemcitabine and paclitaxel. Hypertension was the most reported cardiotoxicity as 30 studies documented this cardiovascular adverse event. Other reported treatment-related cardiotoxicities include arrhythmias, atrial fibrillation, bradycardia, cardiac arrest, cardiac failure, coronary artery disease, heart failure, ischemia, left ventricular dysfunction, myocardial infarction, palpitations, and tachycardia.

**Conclusion:** The findings of this systematic review have provided a better understanding of the possible association between cardiotoxicities and anticancer drugs for NSCLC. Whilst variation is observed across different drug classes, the lack of information available on cardiac monitoring can result in underestimation of this association.

**Systematic Review Registration:** https://www.crd.york.ac.uk/prospero/display_record.php?ID=CRD42020191760, identifier PROSPERO CRD42020191760.

## 1 Introduction

The WHO’s Global Health Estimates reported that lung cancer and heart diseases are two of the major causes of death in the world (World Health Organization, 2020). Due to drug development in cancer therapies and early detection strategies, death rates from cancer have decreased over the last 30 years (Jemal et al., 2010; 2005; Howlader et al., 2010). However, even though survival rates have improved, cardiovascular (CV) disease has become the second leading cause of long-term morbidity and fatality among cancer survivors (DeSantis et al., 2014; Bodai, 2019). Therefore, the risk of cardiotoxicity is one of the major limitations of oncology drug development, due to drug-induced cardiotoxic complications (Csapo and Lazar, 2014).

According to the GLOBOCAN 2020 database released by the International Agency for Research on Cancer (IARC), it was estimated that there were 19.3 million new cancer cases and 10 million cancer deaths worldwide in 2020 alone (Ferlay et al., 2020). In recent years, there has been a breakthrough in the development of novel targeted oncology drugs. According to the Global Oncology Trends 2021, 17 new oncology therapeutic drugs were launched in 2020 alone for 22 different applications with capmatinib being the first therapy approved for targeting metastatic non-small cell lung cancer (NSCLC) with mesenchymal-epithelial transition (MET) exon 14 skipping while both pralsetinib and selpercatinib approved for rearranged during transfection (RET)-altered NSCLC (IQVIA, 2021).

Cardio-oncology is a field that focuses on the CV diseases in cancer patients and addresses the prevention, diagnosis and treatment of cardiotoxicity brought about by oncology drugs or radiotherapy. Chemotherapy aims to destroy the maximum number of tumour cells with minimal damage to other healthy tissues. However, this can be difficult to achieve due to the non-selectivity of chemotherapeutics (Bursác, 2018). Cardiotoxicity can occur during any stage of the cancer treatments and it includes, but is not limited to, subclinical myocardial toxicity, ischemia, hypertension, supraventricular and ventricular arrhythmias, systolic and diastolic cardiac dysfunction, coronary artery disease and heart failure (Hahn et al., 2014; Ewer and Ewer, 2015; Curigliano et al., 2016). Cardiotoxicity was first observed in 1967 in treating leukaemia patients with daunomycin (a type of anthracycline) (Tan et al., 1967). More reports on cardiotoxicity induced by anthracycline emerged in the early 1970s. Thereafter, there has been an increasing number of reports of cardiotoxicity induced by different oncology drugs, e.g., trastuzumab, cyclophosphamide and ifosfamide (Gollerkeri et al., 2001; Moslehi, 2016).

Cardiotoxicity can be generally defined in two ways, according to time of onset or mechanisms. Based on the time cardiotoxicity occurs after receiving chemotherapy, it can be divided into acute (during and up to 2 weeks after chemotherapy), subacute (2–4 weeks after chemotherapy) and chronic (more than 4 weeks after the completion of course) (Bursác, 2018). Chronic cardiotoxicity can be further divided into two types: early onset (cardiotoxicity developing within the first year after chemotherapy); and late onset (cardiotoxicity developing years after the completion of chemotherapy). Initially, there are two types of cardiotoxicity when categorised by mechanisms—Type I is often caused by anthracyclines and chemotherapeutics, of irreversible cardiac cells death and is related to cumulative dosage; while Type II is usually caused by biological or target therapy, of reversible cells dysfunction and is not dose related (Bursác, 2018). Although Type I versus Type II cardiotoxicity was originally described, increasingly more nuanced mechanisms and types of cardiotoxicity have been identified (Tocchetti et al., 2019).

Existing studies suggested that different oncology drugs, even within the same class of drugs, demonstrate different cardiotoxicity potential (Kerkelä et al., 2006; Santoni et al., 2017; Shah et al., 2018). For instance, by blocking the activity of tyrosine kinase, nintedanib prevents the formation of collagen and other extracellular matrix components in the heart, which can lead to cardiotoxicity. In addition, nintedanib may also act directly on the heart, leading to cardiotoxicity. It is believed that the drug can increase the activity of the Na^+^/K^+^-ATPase enzyme, which can lead to a decrease in cardiac output. This decrease in cardiac output can lead to arrhythmias, myocardial infarction, decreased contractility, and even heart failure (Ameri et al., 2021). Both sunitinib and sorafenib are in the same class as nintedanib, but they are believed to induce vascular endothelial growth factor receptors (VEGFR) inhibition, which lead to a decreased production of the vasorelaxant nitric oxide by endothelial cells, thus resulting in hypertension (Wu et al., 2008; León-Mateos et al., 2015).

There are many studies on complications, including cardiotoxicity, relating to thoracic surgery and radiotherapy complications, however there is much less research on the clinical and prognostic impact of toxicity of systemic therapy in non-small cell lung cancer (Zaborowska-Szmit et al., 2020). Therefore, this systematic review aimed to investigate associations between oncology drugs used in the treatment of NSCLC and cardiotoxicity. It also investigated whether different classes of drugs, e.g., anthracyclines, alkylating agents, angiogenesis inhibitors, tyrosine kinase inhibitors (TKIs), and monoclonal antibodies, demonstrate different cardiotoxicity potential. In addition, it aimed to examine whether different dosages of the same drug in initial treatment affect the degree of cardiotoxicities and whether accumulated dosage and/or duration of treatments affect the degree of cardiotoxicities.

## 2 Methods

This systematic review followed the guideline recommended in the ‘Preferred Reporting Items for Systematic Review and Meta-Analysis’ 2020 statement (Page et al., 2021a; Page et al., 2021b). A full version protocol of this systematic review has been published on PROSPERO (CRD42020191760) (Chan et al., 2020).

### 2.1 Search strategy

Electronic databases including Cochrane Library, National Cancer Institute (NCI) Database, PubMed, Scopus and Web of Science were searched for articles reporting clinical trials of cytotoxic drugs where cardiotoxicity was being observed in NSCLC patients. ClinicalTrials.gov and the European Union (EU) Clinical Trials Register were also used to search for recently completed trials. The reference lists of retrieved papers were also hand-searched. All databases and registers were searched from the earliest available date up until November 2020. This time frame was chosen given cardiotoxicity was first observed in 1967 with the use of daunomycin in leukaemia patients (Tan et al., 1967) and more reports on cardiotoxicity induced by anthracyclines emerged in the early 1970s. In addition, from 1997 onwards, there has been a rapid development in targeted treatments and immunotherapies.

Two reviewers (SHYC and YK) independently screened all the articles according to the eligibility criteria until the final list of articles to be reviewed was identified. SHYC and YK independently reviewed all final set of identified articles meeting the eligibility criteria. SHYC extracted all data using the agreed template. SS acted as an adjudicator when there was discrepancy between the two independent reviewers.

### 2.2 Eligibility criteria

This review included studies of patients of ≥18 years old with NSCLC and excluded studies of participants whose treatments involved multiple cancers or radiotherapy only. Only completed clinical trials including at least two arms were included. Other types of studies and reports, e.g., observational studies and conference abstracts were excluded. Observational studies were excluded as they are more prone to bias and confounding associated with their study design than that of randomised controlled trials (RCTs). Participants and/or studies without dosage details and duration of treatments were also excluded. Only records reported in English were included.

### 2.3 Search term

(“non-small cell lung cancer”) AND (“chemotherapy” OR “targeted therapy” OR “immunotherapy” OR “cancer treatment” OR “systemic anticancer therapy” OR “anticancer”) AND (“cardiac adverse events” OR “cardiovascular events” OR “cardiotoxicity” OR “drug-related side effects and adverse reactions”).

### 2.4 Data extraction

The standardised data extraction tool from Cochrane Collaboration’s Tool was adopted for data extraction. Data items were collected under three main areas—setting, participants and outcome.

Setting—“Title of Paper”, “Name of Authors”, “Publication Year”, “Reporting Country”, “Aim of Study”, “Primary Objective”, “Secondary Objectives”, “Study Design”, “Unit of Allocation”, “Enrolment Start Date”, “Enrolment End Date”, “Follow-Up End Date”, “Ethics Approval”, “Clinical Trial Identifier/Registration Number”.

Participants—“Population Description”, “Inclusion Criteria”, “Exclusion Criteria’, “Informed Consent”, “Method of Recruitment”, “Total Number of Cluster Groups, “Total Number of Participants”, “Age”, “Sex”, “Severity of Illness”, “Co-Morbidities”, “Subgroups Measured”, “Name of NSCLC Drug”, “Mode of Administration”, “Dosage Details”, “Duration of Treatment”, “Frequency of Treatment” and “Delivery of Treatment”.

Outcome—“Overall Incidence of Cardiotoxicity”, “Type of Cardiotoxicity”, “Incidence of Each Type of Cardiotoxicity” and “Key Conclusion from Authors”.

Data items were repeatedly collected for each individual placebo or treatment arm where relevant. All data items were input into Microsoft Excel^®^, where each row represented one publication. If certain data items were not available within the publication, then the data and results listed under its corresponding clinical trial identifier were cross-checked to complete the data extraction.

### 2.5 Risk of bias in individual studies

The risk of bias assessment in individual studies was carried out according to the guideline listed in Chapter 8 of the Cochrane Handbook for Systematic Reviews of Interventions (Higgins et al., 2022).

The following criteria were assessed –

– Allocation bias: Allocation concealment
– Attrition bias: Incomplete outcome data
–Performance and detection bias: Blinding of participants, Blinding of outcome assessors
– Reporting bias: Selective reporting
– Selection bias: Random sequence generation

## 3 Results

### 3.1 Results of literature search

**FIGURE 1:**
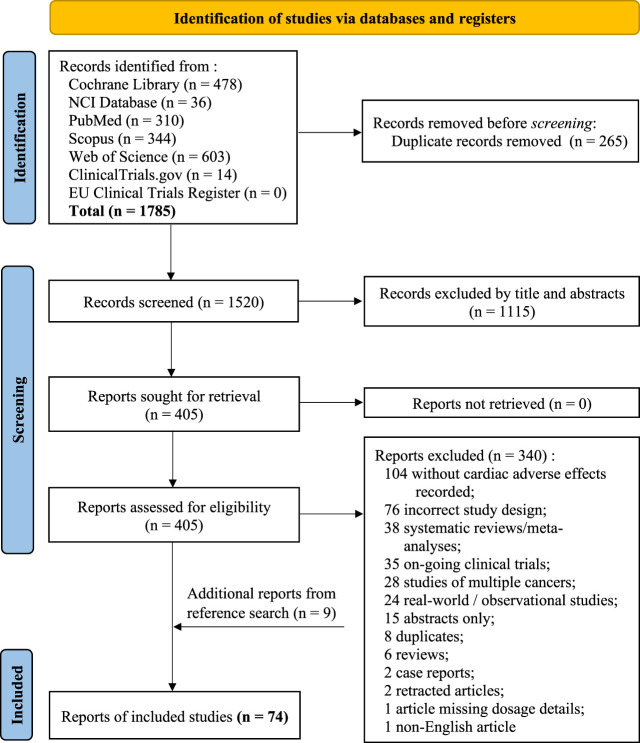
PRISMA 2020 flow diagram for this new systematic review which included searches of databases and registers only.

A total of 1785 records were identified from the seven databases and registers using the search term listed in ‘Methods’. This search time frame (earliest available date up until November 2020) was used in order to maximise the records identified as cardiotoxicity was first observed in 1967 in treating leukaemia patients with daunomycin and more reports on cardiotoxicity induced by anthracycline emerged in the early 1970s. A PRISMA 2020 flow diagram explaining the selection process for this systematic review is presented in Figure 1. A total number of 74 eligible studies were included for data extraction. A summary of the study design, patient population and NSCLC drugs used for all publication is listed in Table 1. Treatment details and patients’ characteristics of each eligible study are available in Supplementary Material S1. Table 2 demonstrates the types of cardiotoxicities and their corresponding number of occurrences reported per publication.

**TABLE 1:** Summary of the study design, patient population and non-small cell lung cancer (NSCLC) drug used for each publication.

References (publication year)	Clinical trial identifier	Reporting country	Study design	Total number of cluster groups	Total number of patients	Age, median (range)	Sex (M/F)	Severity of Illness/NSCLC stage	Co-morbidities	Subgroups measured	Drugs involved
Mizugaki et al. (2015)	NCT01617928	Japan	Open-label, Phase I Study	3	12	67 (44–73 years old)	10M 2F	Stage IIIIB, Stage IV, Postoperative recurrence	Smoker status	Dose	Carboplatin, Paclitaxel, Veliparib
Huang et al. (2020)	NCT03201146	China	Phase I Study	3	12	53.4 (42.2–63.4 years old)	7M 5F	Stage IVA, Stage IVB	Smoker status	Dose	Apatinib, Carboplatin, Pemetrexed
Sebastian et al. (2019)	N/A	Germany & Switzerland	Prospective, multicenter, open-label, uncontrolled phase I/IIa trial	4	46	64.7 (SD: 10.2)	29M 1 7F	Stage IIIB, Stage IV	N/A	Dose	CV9201 (generated using proprietary RNActive^®^ Technology)
Novello et al. (2014a)	NCT01086254	Italy, France, Germany, Spain, United Kingdom	Phase II, randomized, open-label, non-comparative study	2	119	58.7 (29–73 years old)	90M 29F	Stage I, Stage III, Stage IV	Smoker status	N/A	Cisplatin, Iniparib, Gemcitabine
Cappuzzo et al. (2006)	N/A	Italy	Phase II, randomized Study	2	117	72.5 (54–81 years old)	98M 19F	Stage IIIB, Stage IV	N/A	Infusion Duration	Chemotherapy, Gemcitabine
Srinivasa et al. (2020)	N/A	India	Randomized prospective study	2	36	57 (45–65 years old)	33M 3F	Stage IIIA, Stage IIIB	N/A	N/A	Carboplatin, Cisplatin, Etoposide, Paclitaxel
Yoshioka et al. (2017)	NCT01207011	Japan	Randomized, open-label, phase III trial	2	197	20–75 years old	135M 62F	Stage IIIIB, Stage IV, Postoperative recurrence	Smoker status	N/A	Amrubicin, Docetaxel
Johnson et al. (2013)	NCT00257608	United States	Randomized, Double-Blind, Placebo-Controlled, Phase IIIB Trial	2	743	64 (23–88 years old)	389M, 354F	Stage IIIB, Stage IV, Recurrent	Smoker Status	N/A	Bevacizumab, Erlotinib (Chemotherapy prior to trial)
EU Clinical Trials Register. (2011)	MEK114653 (EU Clinicals Register)	France, Greece, Hungary, Italy, Netherlands, South Korea, Spain, United States	Phase II, Open-label, Multicenter, Randomized Study	2	134 (4 drop out)	61.2 (18–64 years old)	69M, 65F	Stage IIIB, Stage IV	N/A	Crossover Phase	Docetaxel, GSK1120212
Gridelli et al. (2001)	N/A	Italy	Pilot Single-Stage Phase II Study	2	98	74 (70–82 years old)	83M, 15F	Stage IIIB, Stage IV	N/A	N/A	Gemcitabine, Vinorelbine
Martoni et al. (1991)	N/A	Italy	Phase I Trial	4	24	60 (36–68 years old)	24M, 0F	Stage IIIB, Stage IV	N/A	Dose, LVEF values	Epirubicin
Sequist et al. (2013), Boehringer Ingelheim (2018a), Wu et al. (2018)	NCT00949650 (LL3)	Argentina, Australia, Austria, Belgium, Brazil, Canada, Chile, France, Germany, Hong Kong, Hungary, Ireland, Italy, Japan, Malaysia, Peru, Philippines, Romania, Russia, South Korea, Taiwan, Thailand, Ukraine, United Kingdom, United States	Global, randomized, open-label phase III study	2	345	60.3 (S.D. 10.1 years old)	121M, 224F	Stage IIIB, Stage IV	Smoker Status	N/A	Afatinib, Cisplatin, Pemetrexed
Boehringer Ingelheim, (2018a) Boehringer Ingelheim. (2018b)	NCT01121393	China, South Korea, Thailand	Randomized, Open-label, Phase III Study	2	364	56.4 (SD: 10.9)	126M, 238F	Stage IIIB, Stage IV	Smoker Status	N/A	Afatinib (BIBW2992), Cisplatin, Gemcitabine
Boehringer Ingelheim (2020)	NCT01466660	Australia, Canada, China, France, Germany, Hong Kong, Ireland, Norway, Singapore, South Korea, Spain, Sweden, Taiwan, United Kingdom	Randomised, Open-label Phase IIB Trial	2	319	62.4 (SD: 11.0)	122M, 197F	Stage IIIB, Stage IV	Smoker Status	N/A	Afatinib, Gefitinib
Hida et al. (2017)	JapicCTI-132316 (Japan Pharmaceutical Information Centre)	Japan	Phase III, Open-label, Multicenter, Randomised Trial	2	207	60.2 (25–85 years old)	82M, 125F	Stage IIIIB, Stage IV, Postoperative recurrence	Smoker Status	N/A	Alectinib, Crizotinib
Berghmans et al. (2013)	NCT00622349	Belgium, France, Greece, Spain	Phase III Trial	3	693	58 (28–84 years old)	523M,170F	Stage IIB, Stage IIIA, Stage IIIB, Stage IV	N/A	N/A	Cisplatin, Docetaxel, Gemcitabine, Ifosfamide
GlaxoSmithKline (2014)	NCT01362296	France, Greece, Hungary, Italy, Netherlands, South Korea, Spain, United States	Phase II, Open-label, Multicenter, Randomised Trial	2	134	61.2 (SD: 9.32)	69M, 65F	Stage IV	Smoker Status	N/A	Docetaxel, GSK1120212 (Trametinib)
Martoni et al. (1999)	N/A	Italy	Pilot Study	2	212	61 (42–72 years old)	179M, 33F	Stage IIIA, Stage IIIB, Stage IV, Recurrence	N/A	N/A	Epirubicin, cisplatinum, vinorelbine
Reck et al. (2015)	NCT00805194	Austria, Belarus, Belgium, Bulgaria, China, Croatia, Czech Republic, Denmark, France, Georgia, Germany, Greece, India, Israel, Italy, Lithuania, Poland, Portugal, Romania, Russia, Slovakia, South Africa, South Korea, Spain, Switzerland, Ukraine, United Kingdom	Randomized, Placebo-Controlled, Phase III trial	2	1314	59.8	955M, 359F	<Stage IIIB, Stage IIIB, Stage IV	Smoker Status	N/A	Docetaxel, Nintedanib
Saito et al. (2003)	N/A	Japan	Parallel	2	25	61.8 (40–79 years old)	16M, 9F	Stage III, Stage IV	N/A	LVEF	Carboplatin, Docetaxel, Paclitaxel
Barlesi et al. (2018)	NCT02395172	Argentina, Australia, Belgium, Brazil, Bulgaria, Chile, Colombia, Croatia, Czech Republic, Denmark, Estonia, France, Hungary, Israel, Italy, Japan, Latvia, Mexico, Peru, Poland, Romania, Russia, Slovakia, South Africa, South Korea, Spain, Switzerland, Taiwan, Turkey, United Kingdom, and United States	Open-label, multicentre, randomised Phase III trial	2	792	63.5 (57–69 years old)	542M 250F	Stage IIIB, Stage IV, Recurrent NSCLC with disease progression after previous platinum doublet treatment>	Smoker Status	N/A	Avelumab, Docetaxel
Camidge et al. (2018)	NCT02737501	Australia, Austria, Canada, Denmark, France, Germany, Hong Kong, Italy, South Korea, Luxembourg, Netherlands, Norway, Singapore, Spain, Sweden, Switzerland, Taiwan, United Kingdom, United States	Open-label, multicenter, randomized, international, Phase III trial	2	275	59 (27–89 years old)	125M 150F	Stage IIIB, Stage IV	Smoker Status	N/A	Brigatinib, Crizotinib
Wachters et al. (2004)	N/A	Netherlands	Randomised phase III trial	2	69	61 (43–76 years old)	49M 20F	Stage IIIA, Stage IIIB Stage IV	N/A	LVEF	Cisplatin, Epirubicin, Gemcitabine
Shaw et al. (2013)	NCT00932893	Australia, Brazil, Bulgaria, Canada, China, France, Germany, Greece, Hong Kong, Hungary, Ireland, Italy, Japan, Netherlands, Poland, Russian Federation, South Korea, Spain, Sweden, Taiwan, United Kingdom, United States	Phase 3, Randomized, Open-label Study	2	347	50 (22–85 years old)	154M 193F	Advanced	Smoker Status	N/A	Crizotinib (PF-02341066), Docetaxel, Pemetrexed
Solomon et al. (2014)	NCT01154140	Australia, Austria, Belgium, Brazil, Canada, Chile, China, Finland, France, Germany, Hong Kong, India, Ireland, Italy, Japan, Luxembourg, Mexico, Netherlands, Norway, Peru, Portugal, Russian Federation, Singapore, South Africa, South Korea, Spain, Switzerland, Taiwan, Ukraine, United Kingdom, United States	Phase 3, Randomized, Open-label Study	2	343	53 (19–78 years old)	131M 212F	Advanced	Smoker Status	N/A	Crizotinib, Carboplatin, Cisplatin, Pemetrexed
Bonomi et al. (2000)	N/A	United States	A Phase III Study	3	574	61.8	365M 209F	Stage IIIB, Stage IV	N/A	Dose	Cisplain, Etoposide, Paclitaxel
Zatloukal et al. (2004)	N/A	Czech Republic	Prospective, randomized open, parallel group study	2	102	61.5 (42–75 years old)	69M 33F	Stage IIIA, Stage IIIB	N/A	N/A	Cisplatin, Vinorelbine
Zarogoulidis et al. (2013)	N/A	Greece	Four-arm Phase III Trial	4	229	62.5	187M 37F	Stage IIIB, Stage IV	Smoker Status	N/A	Bevacizumab, Carboplatin, Docetaxel, Erlotinib
Koch et al. (2011)	NCT00300729	Sweden	Double-blind, placebo-controlled multicentre Phase III Trial	2	316	65.5 (37–85 years old)	160M 156F	Stage IIIB, Stage IV	Smoker Status	N/A	Celecoxib, Chemotherapy (carboplatin/cisplatin/gemcitabine/vinorelbine)
Bi et al. (2019)	NCT01503385	China	A Phase II Randomized Clinical Trial	2	96	60	73M 23F	Stage IIIA, Stage IIIB	Smoker Status	N/A	Celecoxib, Cisplatin, Etoposide
Herbst et al. (2011)	NCT00130728	12 countries including United States	Double-blind, Placebo-Controlled, Randomised Phase 3 trial	2	636	64.9	341M 295F	N/A	Smoker Status	N/A	Bevacizumab, Erlotinib
Kato et al. (2018); Seto et al. (2014)	JapicCTI-111390 (Japan Pharmaceutical Information Centre)	Japan	Open-label, randomised, multicentre, Phase II Study	2	152	67 (59–73 years old)	56M 96F	Stage IIIB Stage IV, Postoperative recurrence	Smoker Status	N/A	Bevacizumab, Erlotinib
National Cancer Institute. (2019)	NCT00126581	United States	A Phase II Randomized, Open label Study	2	181	59 (32–81 years old)	74M 107F	Stage III, Stage IV	Smoker Status	N/A	Carboplatin, Erlotinib, Paclitaxel
Stathopoulos et al. (2004)	N/A	Greece	Multicenter, randomized, phase III trial	2	360	65 (30–84 years old)	312M 48F	Stage IIIA, Stage IIIB, Stage IV	N/A	N/A	Carboplatin, Paclitaxel, Vinorelbine
Valdivieso et al. (1984)	N/A	United States	Prospective, randomised study	2	100	56.5 (33–78 years old)	79M 21F	N/A	Biopsy	Weekly VS. once every 3 weeks doxorubicin	Cisplatin, Cyclo-phosphamide, Doxorubicin, Ftorafur
Baggstrom et al. (2017a)	NCT00693992	United States	Randomized, double-blind, placebo-controlled phase III trial	2	210	64.9 (25–89 years old)	117M 93F	Stage IIIB, Stage IV	Smoker Status	N/A	Chemotherapy, Sunitinib
Paz-Ares et al. (2015)	NCT00863746	Argentina, Austria, Belgium, Brazil, Bulgaria, Canada, Chile, China, France, Germany, Greece, Hong Kong, Hungary, India, Indonesia, Israel, Italy, Japan, South Korea, Netherlands, Pakistan, Peru, Phillippines, Poland, Russia, Singapore, South Africa, Spain, Sweden, Taiwan, Thailand, Turkey, United Kingdom, United States	Phase III, randomized, double-blind, placebo-controlled trial	2	703	≥18 years old	395M 308F	N/A	Smoker Status	N/A	Best supportive care, Sorafenib
Novello et al. (2014b)	NCT00460317	32 countries including Italy, Germany, Romania, Ukraine, United Kingdom, United States	Phase 3, randomized, placebo-controlled, doubleblind study	2	360	60.8 (31–81 years old)	295M 65F	Stage IIIB, Stage IV	Smoker Status	N/A	Carboplatin, Paclitaxel, Motesanib
Akamatsu et al. (2018)	NCT02151981	Australia, Canada, China, France, Germany, Hong Kong, Hungary Italy, Japan, Mexico, Netherlands, Russia, South Korea, Spain, Sweden, Taiwan, United Kingdom, United States	Randomized, open-label, phase III clinical trial	2	419	62.5 (20–90 years old)	150M 269F	N/A	Smoker Status	N/A	Carboplatin, Cisplatin, Pemetrexed, Osimertinib
Kosmidis et al. (2008)	N/A	Greece	Phase III Study	2	452	63 (36–83 years old)	378M 74F	Stage IIIB, Stage IV	N/A	N/A	Carboplatin, Paclitaxel, Gemcitabine
Reinmuth et al. (2019)	NCT02364999	Australia, Brazil, Bulgaria, Chile, Croatia, Czech Republic, France, Germany, Greece, Hungary, India, Italy, Japan, South Korea, Malaysia, Netherlands, Philippines, Poland, Romania, Russia, South Africa, Spain, Taiwan, Thailand, Turkey, Ukraine, United States	Multinational, double-blind, randomized, parallel-group study	2	719	61.5 (25–87 years old)	467M 252F	Stage IIIB, Stage IV, Recurrent	Smoker Status	N/A	Bevacizumab, Carboplatin, Paclitaxel, PF-06439535
Blumenschein et al. (2010)	NCT00094835	United States	Multicenter, Open-label, Dose-finding, Phase IB study of motesanib	3	45	61.3 (32–79 years old)	29M 16F	Stage IIIB, Stage IV	Smoker Status	N/A	Carboplatin, Paclitaxel, Panitumumab, Motesanib
Choy et al. (2013)	NCT00482014	India, United States	Open-label, Randomised Trial	2	98	63.6 (43.7–85.2 years old)	61M 37F	Stage IIIA, Stage IIIB	N/A	N/A	Carboplatin, Cisplatin Pemetrexed
William et al. (2007)	N/A	United States	Open-label, Phase I, Dose-escalationStudy	4	21	52 (38–71 years old)	13M 8F	Stage IV	N/A	Dose	Cisplatin, Docetaxel, Motexafin gadolinium
Chang et al. (1993)	N/A	United States	Phase II Study	3	103	61.3 (31–85 years old)	70M 33F	Stage IV	N/A	N/A	Merbarone, Piroxantrone, Taxol
Kubota et al. (2017)	JapicCTI-121887 (Japan Primary Registries Network)	Japan, South Korea, Hong Kong, Taiwan	Phase III, Randomized, Placebo-Controlled, Double-blind Study	2	401	65 (Upper Quartile: 58; Lower Quartile: 70)	288M 113F	Stage IV, Recurrent	Smoker Status	N/A	Carboplatin, Motesanib, Paclitaxel
Zinner et al. (2015)	NCT00948675	United States	Multicenter, Randomized, Open-label, US-only Phase III Trial	2	361	65.6 (38.4–86.2 years old)	209M 152F	Stage IV	Smoker Status	N/A	Bevacizumab, Carboplatin, Paclitaxel Pemetrexed
Heigener et al. (2013)	NCT00160069	Germany	Prospective, Multicenter, Phase II study	3	128	63	83M 45F	Stage IIIB, Stage IV	N/A	Dose, Duration of Infusion	Sagopilone
Jie Wang et al. (2018)	N/A	China	Randomised Controlled Trial	2	128	No mean/median (36–76 years old)	96M 32F	N/A	N/A	N/A	Cisplatin, Endostar, Pemetrexed
Eli Lilly and Company (2019a)	NCT01469000	China, Japan, South Korea, Taiwan	Multicenter, Randomized, open-label, parallel-arm, phase II study	2	191	61.71 (S.D.: 9.38)	68M 123F	Stage IV	N/A	N/A	Gefitinib, Pemetrexed
Douillard (2004)	N/A	Belgium, Canada, France, Germany, Italy, Spain, United States	Randomized, Double-blind, Placebo-controlled, Phase II Feasibility Study	2	75	61.4	56M 19F	Stage IIIB, Stage IV	N/A	N/A	BMS-275291, Carboplatin, Paclitaxel
Butts et al. (2007)	N/A	Canada, United States	Multicenter, Open-label, Randomized Phase II study	2	131	66 (35–84 years old)	58M 73F	Stage IIIB Stage IV, Recurrent	N/A	N/A	Carboplatin, Cisplatin, Cetuximab, Gemcitabine
Fukuda et al. (2019)	UMIN000008771 (University Hospital Medical Information Network)	Japan	Randomised Phase II Study	2	40	78 (75–83 years old)	23M 17F	Stage IIIB, Stage IV, Postoperative recurrence	Smoker Status	N/A	Bevacizumab, Pemetrexed
Passardi et al. (2008)	N/A	Italy	Randomized Phase II Trial	2	81	63 (35–77 years old)	65M 16F	Stage IV	N/A	N/A	Docetaxel, Gemcitabine
Gatzemeier et al. (2004)	N/A	Canada, Italy, Germany, Netherlands, United Kingdom	Randomized, Open-label, Phase II study	2	101	58.5 (35–76 years old)	63M 38F	Stage IB, Stage IIIB, Stage IV	N/A	N/A	Cisplatin, Gemcitabine, Trastuzumab
Park et al. (2017)	NCT01282151	South Korea	Open-label, Multicenter Prospective Phase III Study	2	148	63.3	103M 45F	Stage IIIB, Stage IV	Smoker Status	N/A	Cisplatin, Docetaxel, Pemetrexed
Movsas et al. (2005)	N/A	Canada, United States	Randomised Trial	2	242	≥18 years old	150M 92F	Stage IIA, Stage IIB, Stage IIIA, Stage IIIB	N/A	N/A	Amifostine, Carboplatin, Paclitaxel
Jänne et al. (2014)	N/A	Canada, Germany, Spain, United States	Randomized, Double-Blind, Phase II Trial	3	200	61.4 (27.8–87.8 years old)	127M 68F	Stage IIIB, Stage IV	Smoker Status	Dose	Cisplatin, Gemcitabine, LY293111
Groen et al. (2011)	N/A	Netherlands	Randomized, Placebo-Controlled Phase III Study	2	561	61 (33–84 years old)	355M 206F	Stage IIIB, Stage IV	N/A	N/A	Carboplatin, Celecoxib, Docetaxel
Currow et al. (2017)	NCT01395914	Australia, Belarus, Belgium, Canada, Czech Republic, France, Germany, Hungary, Israel, Italy, Poland, Russia, Serbia, Slovenia, Spain, Ukraine	Double-blind, safety extension Phase III Study	2	513	62.0	387M 126F	Stage IIIA, Stage IIIB, Stage IV	N/A	N/A	Anamorelin, Placebo
Langer et al. (2017)	NCT00789373	Australia, Belgium, Canada, Finland, France, Germany, Greece, India, Italy, Netherlands, Poland, Portugal, Romania, Spain, Turkey, United Kingdom	Phase 3, Double-Blind, Placebo-Controlled Study	2	939	61.3 (24.4–83.0 years old)	577M 362F	Stage IIIB, Stage IV	Smoker Status	N/A	Cisplatin, Pemetrexed, Placebo
Kotsakis et al. (2015)	NCT00620971	Greece	A Multicenter, Randomized, Phase II study	2	77	59 (36–77 years old)	57M 20F	Stage IIIB, Stage IV	Smoker Status	N/A	Bevacizumab, Cisplatin, Docetaxel, Gemcitabine, Vinorelbine
Eli Lilly and Company (2015)	NCT00112294	United States	A Phase III, Randomised, Open Label Study	2	676	64 (S.D.: 10.2)	396M 280F	N/A	N/A	N/A	Carboplatin, Cetuximab, Taxane (Paclitaxel/Docetaxel)
GlaxoSmithKline (2019)	NCT01868022	Belgium, Denmark, Netherlands, Russia, Spain, United Kingdom, United States	Multi-arm, Non-randomized, Open-Label Phase IB Study	9	65	66.52 (S.D.: 3.08)	52M 13F	Stage IIIB, Stage IV	N/A	Dose	Carboplatin, Cisplatin, Docetaxel, GSK3052230, Paclitaxel, Pemetrexed
Lara et al. (2016)	N/A	United States	Randomised, Phase II Selection Design Trial	2	59	73.1 (40.9–85.9 years old)	24M 35F	Stage IIIB, Stage IV	Smoker Status	N/A	Carboplatin, Erlotinib, Paclitaxel
Wu et al. (2020)	NCT01982955	China, Italy, Malaysia, Singapore, South Korea Spain, Taiwan	Open-label, randomized, Phase 1b/2 study	5	88	N/A	36M 52F	Advanced	N/A	N/A	Carboplatin, Cisplatin, Gefitinib, Pemetrexed, Tepotinib,
Umsawasdi et al. (1989)	N/A	N/A	Randomised Study	2	102	56.5 (33–78 years old)	71M 31F	Stage III	N/A	N/A	Cisplatin, Cyclophosphamide, Doxorubicin
Cortot et al. (2020)	NCT01763671	France	Double-arm, Randomised, Open-label, Multicentre, Phase III Clinical Trial	2	166	59.7 (18.6–81.8 years old)	120M 46F	Stage III, Stage IV	Smoker Status	N/A	Bevacizumab, Docetaxel, Paclitaxel
AstraZeneca (2021)	NCT01933932	Argentina, Australia, Austria, Belgium, Brazil, Bulgaria, Canada, Chile, France, Germany, Hungary, Israel, Italy, Mexico, Netherlands, Peru, Poland, Portugal, Romania, Russian Federation, Spain, Sweden, Turkey, Ukraine, United Kingdom, United States	A Phase III, Double-Blind, Randomised, Placebo-Controlled Study	2	510	61.4 (S.D.: 8.3)	303M 207F	Stage IIIB, Stage IV	N/A	N/A	Docetaxel, Selumetinib
Johnson et al. (2004)	N/A	United States	Randomized Phase II Study	3	99	≥18 years old	60M 39F	Stage IIIB, Stage IV	N/A	Dose	Bevacizumab, Carboplatin, Paclitaxel
Eli Lilly and Company (2022)	NCT00981058	Australia, Austria, Belgium, Brazil, Canada, Croatia, France, Germany, Greece, Hungary, Italy, South Korea, Philippines, Poland, Portugal, Romania, Russia, Serbia, Singapore, Slovakia, South Africa, Spain, Taiwan, Thailand, United Kingdom, United States	Multinational, Randomized, Multicenter, Open-label, Phase III Study	2	1093	62 (32–86 years old)	908M 185F	Stage IIIB, Stage IV	Smoker Status	N/A	Cisplatin, Gemcitabine, Necitumumab
Eli Lilly and Company (2021)	NCT00982111	Australia, Austria, Belgium, Brazil, Canada, Croatia, France, Germany, Greece, Hungary, Italy, Poland, Portugal, Romania, Russia, Slovakia, South Africa, Spain, United Kingdom, United States	Multinational, Randomized, Multicenter, Open-label Phase III Study	2	633	61 (26–88 years old)	424M 209F	Stage IIIB, Stage IV	Smoker Status	N/A	Cisplatin, Pemetrexed, Necitumumab
Eli Lilly and Company (2019b)	NCT01769391	Germany, South Korea, Mexico, Poland, Russia, United States	Randomized, Multicenter, Open-Label, Phase II Study	2	167	65.3	131M 36F	Stage IV	N/A	N/A	Carboplatin, Paclitaxel, Necitumumab

**TABLE 2:** Types of cardiotoxicity and their corresponding number of frequencies reported per publication.

References (publication year)	Drug combination	Dose escalation study	Arrhythmia						Cardiac arrest	Cardiac failure	Cardiotoxicity (Grade 1–4)	Hypertension	Hypotension	Ischaemia	Myocardial infarction	Palpitations	Pericardial effusion	Thromboembolic event (both arterial/venous)	Other cardiovascular event
			Arrhythmia (general)	Atrial/Supra-ventricular arrhythmia		Ventricular arrhythmia													
				Atrial fibrillation	Atrial flutter	Bradycardia	Tachycardia	QT prolongation											
Mizugaki et al. (2015)	Carboplatin, Paclitaxel, Veliparib	V-40 mg										0							
		V-80 mg										2							
		V-120 mg										0							
Huang et al. (2020)	Apatinib, Carboplatin, Pemetrexed	A-750 mg										2							
		A-500 mg										2							
		A-500 mg 2/1 (500 mg/day 2 weeks on 1 week off)										1							
Sebastian et al. (2019)	CV9201	CI - 400 μg					0												
		CII - 800 μg					0												
		CIII - 1600 μg					0												
		Phase IIA - 1600 μg					1												
Novello et al. (2014a)	Cisplatin, Iniparib, Gemcitabine	GC										9							
		GCI										12							
Cappuzzo et al. (2006)	Chemotherapy, Gemcitabine	Standard 50 mg/min									16								
		Low 10 mg/min									11								
Srinivasa et al. (2020)	Carboplatin, Cisplatin, Etoposide, Paclitaxel	Cis-Etop									0								
		Car-Pac									0								
Yoshioka et al. (2017)	Amrubicin, Docetaxel	Amrubicin		1	1											2	1		Ventricular extrasystole: 2 Cardiac tamponade: 1
		Docetaxel		0	0											0	0		
Johnson et al. (2013)	Bevacizumab, Erlotinib (CT prior to trial)	Bev-Plac									31	85							
		Bev-Erlo									29	88							
Gridelli et al. (2001)	Gemcitabine, Vinorelbine	Gem									0								
		Gem-Vin									6								
Martoni et al. (1991)	Epirubicin	120Epi																	LVEF value decrease
		135Epi																	
		150Epi																	
		165Epi											1						
Sequist et al. (2013), Boehringer Ingelheim (2018a), Wu et al. (2018)	Afatinib, Cisplatin, Pemetrexed	Afatinib			0							14	1		0		1	1	Mitral valve incompetence: 1
		Pemetrexed/Cisplatin Chemotherapy			1							14	0		1		0	2	
Boehringer Ingelheim (2018b)	Afatinib, Cisplatin, Gemcitabine	Afatinib								0								0	
		Cisplatin, Gemcitabine Chemotherapy								1								1	
Boehringer Ingelheim (2020)	Afatinib, Gefitinib	Afatinib		0								0			2		1		Acute coronary syndrome: 1 Angina pectoris: 1 Coronary heart disease: 1
		Gefitinib		1								1			0		3		Coronary heart disease: 1 Coronary artery occlusion: 1
Hida et al. (2017)	Alectinib, Crizotinib	Alectinib				1													
		Crizotinib				6													
Berghmans et al. (2013)	Cisplatin, Docetaxel, Gemcitabine, Ifosfamide	IG									10								
		GIP									8								
		DP									20								
EU Clinical Trials Register, (2011); GlaxoSmithKline, (2014)	Docetaxel, GSK1120212 (Trametinib)	Doc										1							
		Tra										13							
Martoni et al. (1999)	Epirubicin, Cisplatinum, Vinorelbine	HDEpi-Cis					3												>15% LVEF decrease: 9
		Vin-Cis					0												>15% LVEF decrease: 3
Reck et al. (2015)	Docetaxel, Nintedanib	Doc-Nin										23						22	
		Doc-Plac										6						19	
Saito et al. (2003)	Carboplatin, Docetaxel, Paclitaxel	Car-Doc									4								
		Car-Pac									2								
Barlesi et al. (2018)	Avelumab, Docetaxel	Avelumab								1									Person with acute cardiac failure also suffered from autoimmune myocarditis
		Docetaxel								0									Cardiovascular insufficiency: 1
Camidge et al. (2018)	Brigatinib, Crizotinib	Brigatinib,				7						31						0	
		Crizotinib				17						10						8	
Wachters et al. (2003)	Cisplatin, Epirubicin, Gemcitabine	Gem-Cis									7				0				
		Gem-Epi									21				1				
Shaw et al. (2013)	Crizotinib, Docetaxel, Pemetrexed	Criz	1			9	0		1					1			1		Cardiac tamponade: 1 Coronary artery disease: 1 Syncope: 1
		Doc-Pem	0			0	1		0					0			2		Cardiac tamponade: 1
Solomon et al. (2014)	Crizotinib, Carboplatin, Cisplatin, Pemetrexed	Criz		1		33			0					0			0		Atrioventricular block: 1 Cardiac tamponade: 2
		Pem-Car/Cis		1		1			1					1			1		Pericarditis: 1 Syncope: 2
Bonomi et al. (2000)	Cisplain, Etoposide, Paclitaxel	Cis-Etop																	Fatal cardiac events: 1
		Cis-250Pac																	Fatal cardiac events: 1
		Cis-135Pac																	Fatal cardiac events: 4
		*The six fatal Grade 5 cardiac events listed above were summarised overall instead of by treatment group - sudden death in 3 patients, myocardial infarction in 2 patients, and hypotension with acute pericarditis in 1 patient																	
Zatloukal et al. (2004)	Cisplatin, Vinorelbine	Con									1								
		Seq									0								
Zarogoulidis et al. (2013)	Bevacizumab, Carboplatin, Docetaxel, Erlotinib	Car-Doc									2								
		Car-Doc-Erlo																	
		Bev-Car-Doc										3							
		Bev-Car-Doc-Erlo										2							
Koch et al. (2011)	Celecoxib, Chemotherapy (carboplatin/cisplatin/gemcitabine/vinorelbine)	Celecoxib												2				17	Cerebrovascular ischaemia: 4
		Placebo												1				12	Cerebrovascular ischaemia: 1
Bi et al. (2019)	Celecoxib, Cisplatin, Etoposide	CE									5								
		CE-Cele									0								
Herbst et al. (2011)	Bevacizumab, Erlotinib	Erlo										4						1	
		Erlo-Bev										15						12	
Seto et al. (2014), Kato et al. (2018)	Bevacizumab, Erlotinib	Erlo									2	11						3	
		Erlo-Bev								1		58						3	
National Cancer Institute, (2019)	Carboplatin, Erlotinib, Paclitaxel	Erlo	1	1		0	1				1	8	0	0		3	0	3	
		Erlo-Car-Pac	1	0		1	4				0	9	6	1		2	1	12	
Stathopoulos et al. (2004)	Carboplatin, Paclitaxel, Vinorelbine	Pac-Car									3								
		Pac-Vin									6								
Valdivieso et al. (1984)	Cisplatin, Cyclo-phosphamide, Doxorubicin, Ftorafur	Weekly-Dox																	
		Standard-Dox																	
		* By an objective grading system of myocardial damage by endomyocardial biopsy, it was suggested that the weekly administration of doxorubicin was associated with lower cardiac toxicity than that of the standard/tri-weekly administration of doxorubicin																	
Baggstrom et al. (2017a)	Chemotherapy, Sunitinib	CT-Placebo										9						1	
		CT- Sunitinib										27						0	
Paz-Ares et al. (2015)	Best supportive care, Sorafenib	BSC-Placebo										16							
		BSC-Sorafenib										68							
Novello et al. (2014b)	Carboplatin, Paclitaxel, Motesanib	Car-Pac-Placebo										15	2						
		Car-Pac-Mote										47	3						
Akamatsu et al. (2018)	Carboplatin, Cisplatin, Pemetrexed, Osimertinib	Osim						7		9									
		Plat (car/cis)-Pem						1		0									
Kosmidis et al. (2008)	Carboplatin, Paclitaxel, Gemcitabine	Gem-Pac									2								
		Gem-Car									1								
Reinmuth et al. (2019)	Bevacizumab, Carboplatin, Paclitaxel, PF-06439535	Car-Pac-Bev								3		32						10	Cardiac disorders: 12
		Car-Pac-PF06439535								1		34						14	Cardiac disorders: 10
Blumenschein et al. (2010)	Carboplatin, Paclitaxel, Panitumumab, Motesanib	Mote(E)-CP		1			0					10							Conduction disorder: 1
		Mote(E)-Pani		1			1					6							
		Mote(125)-CP-Pani		0			0					1							
Choy et al. (2013)	Carboplatin, Cisplatin, Pemetrexed	Pem-Car											2						
		Pem-Cis											4						
William et al. (2007)	Cisplatin, Docetaxel, Motexafin gadolinium	MGd-2.5										0			0				
		MGd-5										1			1				
		MGd-10										1			1				
		MGd-15										2			0				
Chang et al. (1993)	Merbarone, Piroxantrone, Taxol	Merba									3								
		Piro									1								
		Taxol									4								
Kubota et al. (2017)	Carboplatin, Motesanib, Paclitaxel	Car-Pac-Placebo										29							
		Car-Pac-Mote										86							
Zinner et al. (2015)	Bevacizumab, Carboplatin, Paclitaxel, Pemetrexed	Car-Pem										0						0	
		Car-Bev-Pac										4			1			4	
Heigener et al. (2013)	Chemotherapy, Sagopilone	S-16,3 h								0									
		S-22, 0.5 h								1									
		S-22,3 h								0									
Jie Wang et al. (2018)	Cisplatin, Endostar, Pemetrexed	Cis-Pem															40		
		Cis-Pem-Endostar															54		
Eli Lilly and Company (2019a)	Gefitinib, Pemetrexed	Gef		0						1		4	0			1		0	Angine pectoris: 0
		Gef-Pem		1						0		8	1			1		1	Angina pectoris: 1
Douillard (2004)	BMS-275291, Carboplatin, Paclitaxel	Car-Pac-Placebo	0			0	1							2					
		Car-Pac-BMS275291	1			1	0							1					
Butts et al. (2007)	Carboplatin, Cisplatin, Cetuximab, Gemcitabine	Car-Cis-Gem											2						
		Car-Cis-Gem-Cet											2						
Fukuda et al. (2019)	Bevacizumab, Pemetrexed	CT-Pem										0						0	
		CT-Pem-Bev										0						0	
Passardi et al. (2008)	Docetaxel, Gemcitabine	Gem3,8-Doc1									0								
		Gem1,8-Doc8									1								
Gatzemeier et al. (2004)	Cisplatin, Gemcitabine, Trastuzumab	Cis-Gem																	LVEF decrease >15%: 0 LVEF <30%: 0
		Cis-Gem-Tras																	LVEF decrease >15%: 8 LVEF <30%: 0
Park et al. (2017)	Cisplatin, Docetaxel, Pemetrexed	Cis-Doc																1	
		Cis-Pem																2	
Movsas et al. (2005)	Amifostine, Carboplatin, Paclitaxel	Car-Pac									11								
		Car-Pac-Ami									30								
Jänne et al. (2014)	Cisplatin, Gemcitabine, LY293111	Cis-Gem-Placebo																	
		Cis-Gem-200LY																	Cardiorespiratory arrest: 1
		Cis-Gem-600LY																	
Groen et al. (2011)	Carboplatin, Celecoxib, Docetaxel	Car-Doc		0								0					1	0	Pulmonary embolism: 2
		Car-Doc-Celeco		1								1					0	1	Pulmonary embolism: 3
Currow et al. (2017)	Anamorelin, Placebo	Anamorelin									13								Electrocardiogram:4 Ischemic Heart Disease: 4
		Placebo									4								Electrocardiogram:7 Ischemic Heart Disease: 0
Langer et al. (2017)	Cisplatin, Pemetrexed, Placebo	Induction: Cis-Pem	1	1			3		3	1		2		3	3	1	2	2	Acute coronary syndrome: 1 Cardiac tamponade: 1 Cardio-respiratory arrest: 3 Diastolic dysfunction: 1 Pericarditis: 1
		Maintenance: Pem	1	0			0		0	0		0		0	1	0	0	0	Pericarditis: 2 Ventricular fibrillation: 1
		Maintenance: Place	0	0			0		1	0		0		0	0	0	1	0	
Kotsakis et al. (2015)	Bevacizumab, Cisplatin, Docetaxel, Gemcitabine, Vinorelbine	VCB - > DGB										0							
		DCB										1							
Eli Lilly and Company (2015)	Carboplatin, Cetuximab, Taxane (Paclitaxel/Docetaxel)	Tax-Car	1	1	1		7		3				20	0	2		1	5	
		Tax-Car-Cel	1	6	0		27		3				39	1	5		4	3	Cardio-respiratory arrest: 2
GlaxoSmithKline (2019)	Carboplatin, Cisplatin, Docetaxel, GSK3052230, Paclitaxel, Pemetrexed	5GSK-Car-Pac	0	0			0					0	0		0	0	0	0	
		10GSK-Car-Pac	0	0			0					0	0		0	0	0	0	
		20GSK-Car-Pac	0	0			1					2	0		0	0	0	1	Cardiomegaly: 1
		5GSK-Doc	0	0			1					0	0		1	0	1	0	Acute Coronary Syndrome: 1 Angina Pectoris: 1
		10GSK-Doc	0	0			0					0	1		0	0	0	0	
		20GSK-Doc	1	0			0					0	0		0	0	0	0	
		10GSK-Cis-Pem	0	1			0					0	0		0	1	0	0	
		15GSK-Cis-Pem	0	0			0					5	1		0	1	0	0	Conduction disorder: 1
		20GSK-Cis-Pem	0	0			1					2	1		0	0	0	0	Conduction disorder: 1 Left ventricular hypertrophy: 1 Ventricular extrasystoles: 1
Lara et al. (2016)	Carboplatin, Erlotinib, Paclitaxel	Erlo									3								
		Erlo-Car-Pac									3								
Wu et al. (2020)	Carboplatin, Cisplatin, Gefitinib, Pemetrexed, Tepotinib,	1b-300Tep-Gef	0	0													0		
		1b-500Tep-Gef	1	1													1		Cardiac discomfort: 1
		2Neg-Tep-Gef	0	0													0		
		2Neg-Pem-Car/Cis	0	0													0		
		2Pos-Tep-Gef	0	0													0		Supraventricular extrasystoles: 1
Umsawasdi et al. (1989)	Cisplatin, Cyclophosphamide, Doxorubicin	Weekly-Dox																	
		Triweekly-Dox																	
		* Endomyocardial biopsies were done when a total cumulative doxorubicin dose of 300 or 480 mg/m^2^ was reached. Results showed an increase in cardiotoxicity with an increase dosage of doxorubicin, and that the weekly administration of doxorubicin was less toxic than that of the standard/tri-weekly administration of doxorubicin																	
Cortot et al. (2020)	Bevacizumab, Docetaxel, Paclitaxel	Bev-Pac										22							Ischaemic stroke leading to death: 1
		Doc										0							
AstraZeneca (2021)	Docetaxel, Selumetinib	Doc-Plac		4		0	10			2		3	8			0	5	4	Cardiovascular insufficiency: 1 Cardiomegaly: 1
		Doc-Selu		5		2	6			8 (1 is congestive)		4	15			1	0	7	Bundle branch block left: 1 Coronary artery dissection: 1 Diastolic dysfunction: 1 Left Ventricular Dysfunction:1 Mitral valve imcopetence: 1 Pericarditis constrictive: 1
Johnson et al. (2004)	Bevacizumab, Carboplatin, Paclitaxel	Car-Pac										1						3	
		Car-Pac-7.5Bev										5						4	
		Car-Pac-15Bev										6						6	
Eli Lilly and Company (2022)	Cisplatin, Gemcitabine, Necitumumab	Cis-Gem		4	1		0		0	4*			0	1	3		1	4	Acute Coronary Syndrome: 1 Cardio-respiratory arrest: 1 Pericarditis: 1 * including 1 acute, 1 congestive
		Cis-Gem-Nec		3	0		2		2	1*			2	0	4		0	9	Cardiac Tamponade: 1 Cardio-respiratory arrest: 3 Coronary artery disease: 1 * including 1 congestive
Eli Lilly and Company (2021)	Cisplatin, Pemetrexed, Necitumumab	Cis-Pem	0	2	0		1			0		30			1		2	2	Angina pectoris: 1 Cardiomyopathy: 1
		Cis-Pem-Nec	1	2	2		1			2		17			2		3	6	Cardiac tamponade: 2 Cardio-respiratory arrest: 1 Cardiopulmonary failure: 1
Eli Lilly and Company (2019b)	Carboplatin, Paclitaxel, Necitumumab	Car-Pac		4					0	0			7		0			1	
		Car-Pac-Nec		4					1	1*			13		2			1	* including 1 congestive

Remarks: All cardiovascular events with ≤3 studies reported are include in “others”.

Of the 74 eligible studies, 67 reported treatment emergent cardiovascular events, i.e., arrhythmias, atrial fibrillation, bradycardia, cardiac arrest, cardiac failure, coronary artery disease, heart failure, hypertension, ischemia, left ventricular dysfunction, myocardial infarction, palpitations, and tachycardia.

Based on data extracted from the included studies, anticancer drugs for NSCLC that are associated with cardiovascular events include bevacizumab, carboplatin, cisplatin, crizotinib, docetaxel, erlotinib, gemcitabine and paclitaxel.

### 3.2 Dose-related cardiotoxicity

As shown in Table 2, twelve studies reported the use of different or escalating dosages of anticancer drugs.

According to the study by Mizugaki et al., cardiotoxicity, i.e., hypertension, was observed only in the 80 mg veliparib cohort, but neither the 40 mg nor the 120 mg cohort, so it cannot be concluded that veliparib is associated with dose-related cardiotoxicity (Mizugaki et al., 2015).

In the study by Huang M, 2020, patients received oral apatinib combined with intravenous pemetrexed and intravenous carboplatin for 4 cycles. Pemetrexed (500 mg/m^2^) and carboplatin (AUC = 5) were given on day 1 of 21-day cycle. The incidence of hypertension of the cohort which received 500 mg of apatinib per day for 2 weeks and then 1 week off (16.7%) was lower than the other two cohorts which received 500 mg (66.7%) and 700 mg (66.7%) of apatinib per day for 3 weeks respectively (Huang et al., 2020). In the study by Huang M, 2020, patients received oral apatinib combined with intravenous pemetrexed and intravenous carboplatin for 4 cycles. Pemetrexed (500 mg/m^2^) and carboplatin (AUC = 5) were given on day 1 of 21-day cycle. The incidence of hypertension of the cohort which received 500 mg of apatinib per day for 2 weeks and then 1 week off (16.7%) was significantly lower than the other two cohorts which received 500 mg (66.7%) and 700 mg (66.7%) of apatinib per day for 3 weeks respectively.

For CV9201, no dose-limiting toxicity was found across the three cohorts (400 μg, 800 μg, 1600 µg) during the Phase I trial, so 1600 µg was chosen to be used for the Phase II trial. With a larger sample size (*n* = 37), it was reported that one patient suffered from atrial tachycardia, however this adverse event was considered unrelated to the treatment by the clinicians of this trial (Sebastian et al., 2019).

Although reported incidence of cardiotoxicity in Arm A (standard infusion duration 50 mg/min) and Arm B (low infusion duration 10 mg/min) were 28.5% and 18.1% respectively in the study by Cappuzzo et al., it was believed that only one event of cardiac stroke in Arm B was associated with gemcitabine (Cappuzzo et al., 2006).

It was reported in Martoni et al. that 1 of the 3 patients in the cohort who initially received 165 mg/m^2^ dose and later continued the treatment at the reduced dose of 150 mg/m^2^, suffered from severe leukopenia, hypotension and fever after the third course. The patient later died 8 days after the epirubicin dose, which was believed to be caused by septic shock (Martoni et al., 1991). Besides, treatments were discontinued for 4 patients out of the total 24 patients as their left ventricular ejection fraction (LVEF) values dropped by 14%, 20%, 25% and 31% at the cumulative doses of 240 mg/m^2^ (120Epi), 560 mg/m^2^ (120Epi), 300 mg/m^2^ (150Epi) and 516 mg/m^2^ (150Epi) respectively. Despite the drop of LVEF values, no patients experienced any clinical signs of cardiotoxicity either at that time or subsequently. Also, no systematic pattern was observed in decrease of LVEF values across cohorts of different dosage and accumulated dosage, so it cannot be concluded that whether certain single and/or accumulated dosage of epirubicin had possibly caused a decrease in LVEF values (Martoni et al., 1991).

In Bonomi et al., fatal cardiac events were observed in 0.5% (Cis-Etop), 0.5% (Cis-Pac-250) and 2% (Cis-Pac-135) patients respectively. The frequency of cardiotoxicity was significantly higher when using higher dose (250 mg/m^2^) of paclitaxel (*p* = 0.026) whereas that of lower dose (135 mg/m^2^) of paclitaxel was insignificant (*p* = 0.143). Grade 5 cardiac events were also observed in 6 patients, including 3 sudden deaths, 2 myocardial infarction and 1 hypotension with acute pericarditis. However, this data needs to be considered carefully as four of the above-mentioned patients had a history of cardiovascular disease—two patients suffered from coronary artery disease, one patient had hypertension and the remaining was previously treated for cardiac arrhythmia (Bonomi et al., 2000).

A study published by Valdivieso et al., in 1984 demonstrated that the administration of weekly 20 mg/m^2^ of doxorubicin was associated with a lower incidence of cardiotoxicity than that of the standard regimen (every 3 weeks at 60 mg/m^2^ of doxorubicin) (Valdivieso et al., 1984). Cardiotoxicity was determined by an objective grading system of myocardial damage by endomyocardial biopsy. This study’s results aligned with previous studies which also suggested that the weekly treatment schedule was less cardiotoxic (Weiss et al., 1976; Weiss and Manthel, 1977). Due to the reduced risk of cardiotoxicity in weekly schedule of doxorubicin, it was suggested that the cardiotoxicity of doxorubicin was associated with its peak plasma levels (Valdivieso et al., 1984).

Dose-limiting cardiotoxicities were observed in the 10 mg/kg (day 1 only) and 7.5 mg/kg (day 1 and/or day 2) motexafin gadolinium cohorts in William Jr. et al. Four patients suffered from hypertension and two patients suffered from myocardial ischemia within the first 24 hours administration of motexafin gadolinium (William et al., 2007). For the two patients who suffered from myocardial ischaemia—one experienced chest pain during the infusion of cycle 2 docetaxel, while the other patient experienced dyspnea 5 hours after completion of chemotherapy. Cardiac enzyme elevations were observed in both patients; T-wave inversion on the electrocardiogram and non-specific ST segment alterations in the electrocardiogram was observed in respective patient (William et al., 2007).

In Heigener DF et al., one patient, who was treated with 22 mg/m^2^ sagopilone at 0.5 hour infusion every 3 weeks, suffered from cardiac failure. However, it was considered that this was not a dose-limiting factor and also non-related to the drug as this was a single case and the cause of death for other cases were also miscellaneous events (Heigener et al., 2013).

In Jänne. et al., it was reported that there was a treatment-related death caused by cardiorespiratory arrest, which was treated with 200 mg LY293111 with gemcitabine and cisplatin. However, no treatment-related cardiotoxicity was reported in the 600 mg LY293111 cohort (Jänne et al., 2014).

In a non-randomised, 9-arm, open label Phase IB clinical trial which evaluated anticancer activity of GSK3052230, three different combinations of drugs were used—1) GSK3052230 with carboplatin and paclitaxel, 2) GSK3052230 with docetaxel and 3) GSK3052230 with cisplatin and pemetrexed. For each combination, there were three arms which consisted of different dosages of GSK3052230, i.e., 5 mg/kg, 10 mg/kg and 20 mg/kg of GSK3052230 (GlaxoSmithKline, 2019). Counts of cardiotoxicity reported for each individual arm were shown in Table 2. As there was no systematic pattern of cardiotoxicity across arms, so it cannot be concluded that if there was dose-related cardiotoxicity associated with GSK3052230 (GlaxoSmithKline, 2019).

In a clinical trial conducted by Johnson. et al., carboplatin and paclitaxel were used as a control arm, and 2 arms consisted of different dosages of bevacizumab with carboplatin and paclitaxel were investigated. It was reported that higher dosage (15 mg/kg) of bevacizumab experienced a higher incidences of cardiotoxicity than that of 7.5 mg/kg of bevacizumab (Johnson et al., 2004).

### 3.3 Risk of bias assessment

Risk of bias assessment is important as it can provide insight of possible bias for each study, thus aiding the transparency of results and findings in this systematic review. Table 3 includes a summary of the risk of bias assessment of each individual study. Light gray (+) indicates low risk; dark gray (−) indicates high risk and medium gray (?) means unclear as there is not enough information to make a clear judgement.

**TABLE 3:** A summary of the risk of bias assessment of all eligible studies.

References (publication year)	Random sequence generation (selection bias)	Allocation concealment (selection bias)	Blinding of participants and personnel (performance bias)	Blinding of outcome assessment (detection bias)	Incomplete outcome data (attrition bias)	Selective reporting (reporting bias)	Other bias
Mizugaki et al. (2015)	?	–	–	–	+	+	?
Huang et al. (2020)	?	?	?	?	+	+	?
Sebastian et al. (2019)	?	?	?	?	+	+	?
Novello et al. (2014a)	?	+	?	?	+	+	?
Cappuzzo et al. (2006)	?	?	?	?	+	?	–
Srinivasa et al. (2020)	?	?	?	?	+	+	?
Yoshioka et al. (2017)	+	–	–	–	+	+	?
Johnson et al. (2013)	+	+	+	+	+	+	+
EU Clinical Trials Register. (2011)	?	+	?	?	+	+	?
Gridelli et al. (2001)	+	+	?	?	+	+	?
Martoni et al. (1991)	?	?	–	–	+	+	?
Sequist et al. (2013), Boehringer Ingelheim (2018a), Wu et al. (2018)	+	+	–	–	+	+	?
Boehringer Ingelheim (2018b)	+	+	–	–	+	+	?
Boehringer Ingelheim (2020)	+	+	–	–	+	+	?
Hida et al. (2017)	+	–	–	–	+	+	?
Berghmans et al. (2013)	+	+	?	?	+	+	?
GlaxoSmithKline (2014)	+	+	+	+	+	+	+
Martoni et al. (1999)	+	+	–	–	+	+	?
Reck et al. (2015)	?	?	+	?	+	+	?
Saito et al. (2003)	?	?	?	?	+	+	?
Barlesi et al. (2018)	+	+	–	?	+	+	?
Camidge et al. (2018)	+	?	?	?	+	+	?
Wachters et al. (2004)	+	?	?	?	+	+	?
Shaw et al. (2013)	–	–	–	–	+	+	?
Solomon et al. (2014)	–	–	–	–	+	+	?
Bonomi et al. (2000)	+	?	?	?	+	+	?
Zatloukal et al. (2004)	+	?	–	–	+	+	?
Zarogoulidis et al. (2013)	+	?	?	?	+	+	?
Koch et al. (2011)	+	+	+	+	+	+	+
Bi et al. (2019)	+	+	–	–	+	+	?
Herbst et al. (2011)	+	+	+	+	+	+	+
Seto et al. (2014)	+	+	–	+	+	+	?
(National Cancer Institute (NCI), 2019)	+	+	+	+	+	+	+
Kato et al. (2018)	+	+	–	+	+	+	?
Stathopoulos et al. (2004)	+	–	?	?	+	+	?
Valdivieso et al. (1984)	+	?	?	?	+	+	?
Baggstrom et al. (2017a)	+	–	?	?	+	+	?
Paz-Ares et al. (2015)	+	–	+	?	+	+	?
Novello et al. (2014b)	+	+	+	?	+	+	?
Akamatsu et al. (2018)	+	+	–	–	+	+	?
Kosmidis et al. (2008)	+	+	–	?	+	+	?
Reinmuth et al. (2019)	+	+	+	?	+	+	?
Blumenschein et al. (2010)	?	?	–	–	+	+	?
Choy et al. (2013)	+	?	–	–	+	+	?
William et al. (2007)	?	?	–	–	+	+	?
Chang et al. (1993)	+	?	?	?	+	+	?
Kubota et al. (2017)	+	–	+	+	+	+	?
Zinner et al. (2015)	+	–	?	?	+	+	?
Heigener et al. (2013)	+	?	–	–	+	+	?
Jie Wang et al. (2018)	+	?	?	?	+	+	?
Eli Lilly and Company (2019a)	+	+	+	+	+	+	+
Douillard (2004)	+	?	+	?	+	+	?
Butts et al. (2007)	+	–	–	+	+	+	?
Fukuda et al. (2019)	+	–	?	?	+	?	?
Passardi et al. (2008)	+	?	?	?	+	?	?
Gatzemeier et al. (2004)	+	?	–	?	+	?	?
Park et al. (2017)	+	?	?	?	+	?	?
Movsas et al. (2005)	+	+	+	+	+	+	+
Jänne et al. (2014)	+	?	?	?	+	+	?
Groen et al. (2011)	+	?	?	?	+	?	?
Currow et al. (2017)	+	?	+	+	+	?	?
Langer et al. (2017)	+	?	?	?	+	?	?
Kotsakis et al. (2015)	+	?	–	–	+	?	?
Eli Lilly and Company (2015)	+	+	+	+	+	+	+
GlaxoSmithKline (2019)	+	+	–	+	+	+	+
Lara et al. (2016)	+	?	–	?	+	?	?
Wu et al. (2020)	+	+	+	+	+	?	?
Umsawasdi et al. (1989)	+	?	–	?	+	?	?
Cortot et al. (2020)	+	+	–	?	+	+	?
AstraZeneca (2021)	+	+	+	+	+	+	+
Johnson et al. (2004)	+	+	–	+	+	+	?
Eli Lilly and Company (2022)	+	+	+	+	+	+	+
Eli Lilly and Company (2021)	+	+	+	+	+	+	+
Eli Lilly and Company (2019b)	+	+	+	+	+	+	+

It was observed that for most publications, the risk of blinding of outcome assessment were unclear. Hence, there should be a more comprehend guideline for developing and reporting clinical trials, so to ensure clinical trials are conducted in a manner with as little bias as possible.

## 4 Discussion

Cardiotoxicity is a type of cardiovascular side effect caused by anticancer drugs used to treat NSCLC. This type of toxicity occurs when the anticancer drugs damage the heart or its surrounding structures, leading to a range of symptoms including arrhythmias, congestive heart failure, and high blood pressure. While the risk of cardiotoxicity is low in patients with early-stage NSCLC, it is higher in those with advanced or metastatic cancer. There are several factors that can increase the risk of cardiotoxicity in those receiving NSCLC treatments, such as age, pre-existing heart conditions, and the specific drug(s) used. Certain NSCLC drugs are more likely to cause cardiotoxicity than others, and certain combinations of drugs may also increase the risk. For example, traditional chemotherapy agents including gemcitabine, cisplatin, and carboplatin are all known to cause cardiotoxicity in some patients. With the rapid development of targeted therapies and immunotherapies, it was observed among the included eligible studies that a lot of treatments were still used in combination with conventional treatments, such as cisplatin, carboplatin, docetaxel and paclitaxel. Similar findings was reported by other literature, in which cytotoxic chemotherapies are still being used in ∼30% of cancer regiments (McGowan et al., 2017). Table 4 categorised all NSCLC drugs included in this systematic review by their therapeutic class, according to ATC/DDD Index 2022 (WHOCC, 2022).

**TABLE 4:** Anticancer drugs included in this systematic review, categorised by therapeutic class.

**Chemotherapy**		**Targeted Therapy**			**Immunotherapy**	
**Anthracycline**	**Platinum Compound**	**Anaplastic Lymphoma Kinase (ALK) Inhibito**r	**Angiogenesis Inhibitor**	**Epidermal Growth Factor Receptor (EGFR) Inhibitor**	**Programmed cell death protein 1/death ligand 1 (PD-1/PDL-1) Inhibitor**	**Epidermal Growth Factor Receptor (EGFR) Inhibitor**
Amrubicin (L01DB10)	Carboplatin (L01XA02)	Alectinib (L01ED03)	Nintedanib (L01EX09)	Erlotinib (L01EB02)	Avelumab (L01FF04)	Cetuximab (L01FE01)
Epirubicin (L01DB03)	Cisplatin (L01XA01)	Brigatinib (L01ED04)	Sorafenib (L01EX02)	Gefitinib (L01EB01)		Necitumumab (L01FE03)
		Crizotinib (L01ED01)	Sunitinib (L01EX01)	Osimertinib (L01EB04)		Panitumumab (L01FE02)

**Alkylating Agent**	**Anti-metabolite Agent**	**Cyclooxygenase-2 (COX-2) Inhibitor**	**Mitogen-activated protein kinase (MEK) inhibitors**	**Poly (ADP-ribose) polymerase (PARP) inhibitor**	**Human Epidermal Growth Factor Receptor 2 (HER2) Inhibitor**	**mRNA-based**
Ifosfamide (L01AA06)	Gemcitabine (L01BC05 - Pyrimidine analogues)	Celecoxib (L01XX33)	Selumetinib (L01EE04)	Veliparib (L01XK05)	Trastuzumab (L01FD01)	CV9201
	Pemetrexed (L01BA04 – folic acid analogues)		GSK1120212/ Trametinib (L01EE01)			

**Plant Alkaloid**		**Vascular Endothelial Growth Factor (VEGFR) Inhibitor**				
Docetaxel (L01CD02)		Bevacizumab (L01FG01)				
Etoposide (L01CB01)						
Paclitaxel (L01CD01)						
Vinorelbine (L01CA04)						

Hypertension was observed in over 30 studies, making it the most reported cardiotoxicity. Hypertension is mostly acute and self-limited and is known to be one of the common non-hematologic adverse events of antiangiogenic agents (Li et al., 2013). This systematic review also found that other drug classes such as anti-microtubule agents, alkylating agents were associated with treatment-induced hypertension which aligns with findings by Chung et al. (Chung et al., 2020). Hypertension was also observed with the combination use of cisplatin, docetaxel and motexafin gadolinium; they were normally observed within the first 24 hours administration of motexafin gadolinium, and subsided after receiving oral clonidine (William et al., 2007).

As most studies reported cardiotoxicity at aggregate level, it is unclear whether certain patient experienced more than one type of cardiotoxicity, therefore it cannot be determined to what extent hypertension could have potentially contributed to other cardiovascular diseases, such as ischaemia in individual patients. Hence, the lack of information available may result in overestimation of the association between NSCLC drugs and cardiotoxicity.

Anthracyclines are effective anticancer treatments, however, their benefits are often limited by possible fatal dose-dependent cardiotoxicity (Smith et al., 2010). Anthracyclines, such as doxorubicin, are believed to cause direct damage to the heart by inducing oxidative stress and direct damage to the cardiomyocytes (Zhang et al., 2012). According to an included study by Valdivieso et al., higher dose of doxorubicin leads to a higher incidence of cardiotoxicity (Valdivieso et al., 1984). This finding was supported by Swain et al., which suggested the incidence of heart failure after doxorubicin treatment increased with cumulative dose (Swain et al., 2003). An included study by Wachters et al., suggested that epirubicin caused a much higher incidence of cardiotoxicity than that of cisplatin (Wachters et al., 2004). In a study by Martoni et al., it was discovered that a higher dose of epirubicin was linked to a higher decrease in LVEF values, but no systematic pattern was observed in decrease of LVEF values across cohorts of different dosage and accumulated dosage, so it cannot be concluded that whether certain single and/or accumulated dosage of epirubicin possibly caused a decrease in LVEF values (Martoni et al., 1991). But this assumption can be supported by other studies, which concluded that epirubicin was associated with cumulative-dose cardiotoxicity (Wils et al., 1990; Feld et al., 1992; Smit et al., 1992). Others, such as daunorubicin, are believed to cause indirect damage to the heart by interfering with calcium homeostasis. One of the potential mechanisms of anthracycline cardiotoxicity is the inhibition of topoisomerase, which causes mitochondrial dysfunction, leading to the activation of cell death pathways and generation of reactive oxygen species (Carrasco et al., 2021). Additionally, different anthracyclines may have different levels of cardiotoxicity due to the presence of different metabolites or active forms of the drug, which could also contribute to the different onset of cardiotoxicity. For anti-microtubule agents, mechanisms of onset of cardiotoxicity include interfering with the normal function of the heart’s cells, such as the contractility of the cells and the electrical conduction pathways; blocking the formation of new microtubules, which is necessary for the heart’s cells to divide and multiply; and direct damage to the heart tissue, leading to arrhythmias, heart failure, and other cardiotoxic effects (Zhang et al., 2019).

Cisplatin is a type of alkylating agents and is also a commonly used drug to treat NSCLC (Table 4). As listed in Table 2, several studies demonstrated that cisplatin can cause cardiotoxicity, which ranged from arrhythmias, hypertension, myocardial infarction to chronic heart failure (Gatzemeier et al., 2004; Wachters et al., 2004; Butts et al., 2007; Berghmans et al., 2013; Choy et al., 2013; Novello et al., 2014a; Jänne et al., 2014, p. 4; Park et al., 2017; Jie Wang et al., 2018; Srinivasa et al., 2020; Eli Lilly and Company, 2022; 2021). The cisplatin-induced cardiotoxicities are possibly related to the imbalance of electrolytes (Miller et al., 2010; Oun and Rowan, 2017). Increased platelet reactivity by activation of arachidonic pathway is believed to be one of the mechanisms of cardiotoxicity caused by alkylating drugs. Oxidative stress and direct endothelial capillary damage with resultant extravasation of proteins, erythrocytes, and toxic metabolites, can then damage the myocardium, leading to cardiomyocyte degeneration and necrosis (Mudd et al., 2021).

For angiogenesis inhibitors that interfere with the vascular endothelial growth factor (VEGF) pathway, such as bevacizumab, can lead to hypertension, cardiac arrhythmias, and congestive heart failure. Bevacizumab is a targeted therapy that starves tumours by preventing new blood vessels from growing. It was observed among a number of eligible studies that there were higher incidence rates of hypertension with the addition of bevacizumab in anticancer treatments than those without. Several studies showed that with the addition of bevacizumab, there was an increased incidence of arterial thromboembolic events. This result was expected as arterial thromboembolism is a known adverse reaction to bevacizumab (Herbst et al., 2011; Johnson et al., 2013; Kato et al., 2018; Reinmuth et al., 2019). These adverse events were potentially caused by the VEGFR inhibition effects of bevacizumab, which negatively affected the coagulation system (Reck et al., 2015). Same as bevacizumab, sorafenib and sunitinib are also angiogenesis inhibitors, and more specifically VEGF receptor kinase inhibitor and multitargeted RTK inhibitors respectively. The mechanism of this class of drug is to inhibit neovascularization which will then inhibit the growth of tumour as new blood vessels are needed for tumours to grow. Sorafenib and sunitinib demonstrated similar cardiotoxicity potentials as only hypertension was observed in both of them (Paz-Ares et al., 2015; Baggstrom et al., 2017b). In contrast, inhibitors of the fibroblast growth factor (FGF) pathway can lead to cardiomyopathy and increased risk of ischemic events due to increased myocardial oxygen consumption. Other angiogenesis inhibitors can cause cardiomyopathy due to their direct effect on the myocardium, leading to decreased contractility (Maurea et al., 2016; Dobbin et al., 2021).

In Gatzemeier et al., it was reported that cardiotoxicity was associated with the use of trastuzumab (Gatzemeier et al., 2004). This clinical finding differed from the safety profile of preclinical studies as there was no evidence of neither acute nor dose-related cardiotoxicity (Mellor et al., 2011). Inhibition of the NRG-1/ErbB2 signalling—a protective intracellular signalling pathway—is one of the proposed mechanisms that causes trastuzumab-induced cardiotoxicity (Perez and Rodeheffer, 2004). It was reported in Barlesi et al. that the patient in the avelumab group with acute cardiac failure also suffered from autoimmune myocarditis (Barlesi et al., 2018). In Butts et al., it was demonstrated that the addition of cetuximab to platinum/gemcitabine treatment did not increase cardiotoxicity as both groups reported the same percentage of cardiovascular events (Butts et al., 2007).

Through this systematic review, it is suggested that several NSCLC treatments are associated with cardiotoxicity, but the actual incidence of cardiotoxicity induced by NSCLC treatments is still undefined. This is because systematic cardiac monitoring was not carried out in most of the clinical trials, thus compromising the ability to detect cardiotoxicity during clinical trials. Moreover, all included clinical trials had different eligibility criteria, treatment regimens and reporting styles, therefore the lack of standardisation made it difficult to compare the safety data among different clinical trials.

In addition, most treatments reported were a combination of several anticancer drugs, hence it was difficult to identify exactly which drug contributes to cardiotoxicity or if a single drug has higher cardiotoxic potential.

This systematic review analysed data collected from clinical trials (i.e., aggregate data instead of individual patients’ data), hence it was difficult to tell whether one person suffered from more than one type of cardiotoxicities. Also, based on the eligibility criteria, some of the studies which did not match the required study design (i.e., single arm study) were excluded even though counts of cardiotoxicity were recorded, so this might have caused selection bias of studies. In addition, the authors of some included publications mentioned that the incidences of cardiotoxicity were believed to be unrelated to the anticancer treatments. Therefore, for this systematic review, we adopted their opinions and did not include those cardiotoxicities thought not to be associated with NSCLC treatments. Moreover, due to the limitations of the eligibility criteria, the drugs included in the eligible studies might not necessarily be the most commonly used first/second-line treatments of NSCLC. Another limitation was that differences in duration of follow-up period among studies may potentially result in inaccurate representation of the frequency of cardiotoxicity associated with corresponding anticancer drug. In some studies, only adverse events with an overall incidence of ≥10% were reported, thus might cause reporting bias. One of the limitations observed was that most cardiotoxicities reported were symptomatic cardiotoxicities, whereas some expected asymptomatic cardiotoxicities such as QT prolongation were not commonly reported, thus it is suggested that systematic cardiac monitoring should be carried out and corresponding data should be reported. Lastly, by restricting our literature search only to studies reported in English other relevant studies might have been missed.

## 5 Conclusion

In the last few decades, there has been a rapid development in cancer therapies and improved detection strategies, hence the death rates caused by cancer have decreased. However, it has been reported that cardiovascular disease has become the second leading cause of long-term morbidity and fatality among cancer survivors. The findings of this systematic review have provided a better understanding of the types of cardiotoxicities each anticancer drug is associated with. However, as systematic cardiac monitoring was not carried out in most of the clinical trials, the actual incidence of cardiotoxicity induced by NSCLC treatments remains undefined. Cardiotoxicity reported ranges from hypertension to heart failure with hypertension being the most common contributor. Although some cardiac adverse events are reversible, further research on identifying patients at risk for potentially serious cardiovascular events as well as implementation of early detection and screening strategies are needed to improve benefit-risk balance of treatments in cancer patients.

## Data Availability

The original contributions presented in the study are included in the article/Supplementary Material, further inquiries can be directed to the corresponding author.

## Supplementary material

Supplementary Table S1 Summary of treatment details and patients’ characteristics of each publication.

## References

[B1] Akamatsu H. Katakami N. Okamoto I. Kato T. Kim Y. H. Imamura F. (2018). Osimertinib in Japanese patients with EGFR T790M mutation-positive advanced non-small-cell lung cancer: AURA3 trial. Cancer Sci. 109, 1930–1938. 10.1111/cas.13623 29697876 PMC5989837

[B2] Ameri P. Tini G. Spallarossa P. Mercurio V. Tocchetti C. G. Porto I. (2021). Cardiovascular safety of the tyrosine kinase inhibitor nintedanib. Br. J. Clin. Pharmacol. 87, 3690–3698. 10.1111/bcp.14793 33620103

[B3] AstraZeneca (2021). “A phase III, double-blind, randomised, placebo-controlled study to assess the efficacy and safety of selumetinib (AZD6244; ARRY-142886) (Hyd-Sulfate) in combination with docetaxel,” in Patients receiving second line treatment for KRAS mutation-positive locally advanced or metastatic non small cell lung cancer (stage IIIB - IV) (SELECT 1) (clinical trial registration No. NCT01933932) (clinicaltrials.gov).

[B4] Baggstrom M. Q. Socinski M. A. Wang X. F. Gu L. Stinchcombe T. E. Edelman M. J. (2017a). Maintenance sunitinib following initial platinum-based combination chemotherapy in advanced-stage IIIB/IV non–small cell lung cancer: A randomized, double-blind, placebo-controlled phase III study—CALGB 30607 (alliance). J. Thorac. Oncol. 12, 843–849. 10.1016/j.jtho.2017.01.022 28161554 PMC5500219

[B5] Baggstrom M. Q. Socinski M. A. Wang X. F. Gu L. Stinchcombe T. E. Edelman M. J. (2017b). Maintenance sunitinib following initial platinum-based combination chemotherapy in advanced-stage IIIB/IV non-small cell lung cancer: A randomized, double-blind, placebo-controlled phase III study-CALGB 30607 (alliance). J. Thorac. Oncol. Off. Publ. Int. Assoc. Study Lung Cancer 12, 843–849. 10.1016/j.jtho.2017.01.022 PMC5500219 28161554

[B6] Barlesi F. Vansteenkiste J. Spigel D. Ishii H. Garassino M. de Marinis F. (2018). Avelumab versus docetaxel in patients with platinum-treated advanced non-small-cell lung cancer (JAVELIN lung 200): An open-label, randomised, phase 3 study. Lancet Oncol. 19, 1468–1479. 10.1016/S1470-2045(18)30673-9 30262187

[B7] Berghmans T. Lafitte J.-J. Scherpereel A. Paesmans M. Lecomte J. Marco V. G. (2013). An ELCWP phase III trial comparing ifosfamide and cisplatin regimens in advanced NSCLC. Anticancer Res. 33, 5477–5482. 24324084

[B8] Bi N. Liang J. Zhou Z. Chen D. Fu Z. Yang X. (2019). Effect of concurrent chemoradiation with celecoxib vs concurrent chemoradiation alone on survival among patients with non–small cell lung cancer with and without cyclooxygenase 2 genetic variants: A phase 2 randomized clinical trial. JAMA Netw. Open 2, e1918070. 10.1001/jamanetworkopen.2019.18070 31851351 PMC6991217

[B9] Blumenschein G. R. Reckamp K. Stephenson G. J. O’Rourke T. Gladish G. McGreivy J. (2010). Phase 1b study of motesanib, an oral angiogenesis inhibitor, in combination with carboplatin/paclitaxel and/or panitumumab for the treatment of advanced non-small cell lung cancer. Clin. Cancer Res. 16, 279–290. 10.1158/1078-0432.CCR-09-1675 20028752

[B10] Bodai B. Tuso P. (2019). Breast cancer survivorship: A comprehensive review of long-term medical issues and lifestyle recommendations. Perm. J. 19, 48–79. 10.7812/TPP/14-241 PMC4403581 25902343

[B11] Boehringer Ingelheim (2018a). A randomised, open-label, Phase III study of BIBW 2992 versus chemotherapy as first-line treatment for patients with stage IIIB or IV adenocarcinoma of the lung harbouring an EGFR activating mutation (clinical trial registration No. NCT00949650). clinicaltrials.gov.

[B12] Boehringer Ingelheim (2018b). LUX-lung 6: A randomized, open-label, Phase III study of BIBW 2992 versus chemotherapy as first-line treatment for patients with stage IIIB or IV adenocarcinoma of the lung harbouring an egfr activating mutation (clinical trial registration No. NCT01121393). clinicaltrials.gov.

[B13] Boehringer Ingelheim (2020). LUX-lung 7: A randomised, open-label Phase IIb trial of afatinib versus gefitinib as first-line treatment of patients with egfr mutation positive advanced adenocarcinoma of the lung (clinical trial registration No. NCT01466660). clinicaltrials.gov.

[B14] Bonomi P. Kim K. Fairclough D. Cella D. Kugler J. Rowinsky E. (2000). Comparison of survival and quality of life in advanced non-small-cell lung cancer patients treated with two dose levels of paclitaxel combined with cisplatin versus etoposide with cisplatin: Results of an eastern cooperative oncology group trial. J. Clin. Oncol. Off. J. Am. Soc. Clin. Oncol. 18, 623–631. 10.1200/JCO.2000.18.3.623 10653877

[B15] Bursác D. (2018). Cardiotoxicity of first-line chemotherapy in patients with advanced non-small cell lung cancer. J. Biodivers. Endanger. Species 9, 1–4. 10.4172/2329-9517.1000345

[B16] Butts C. A. Bodkin D. Middleman E. L. Englund C. W. Ellison D. Alam Y. (2007). Randomized phase II study of gemcitabine plus cisplatin or carboplatin [corrected], with or without cetuximab, as first-line therapy for patients with advanced or metastatic non small-cell lung cancer. J. Clin. Oncol. 25, 5777–5784. 10.1200/JCO.2007.13.0856 18089875

[B17] Camidge D. R. Kim H. R. Ahn M.-J. Yang J. C.-H. Han J.-Y. Lee J.-S. (2018). Brigatinib versus crizotinib in ALK-positive non-small-cell lung cancer. N. Engl. J. Med. 379, 2027–2039. 10.1056/NEJMoa1810171 30280657

[B18] Cappuzzo F. Novello S. De Marinis F. Selvaggi G. Scagliotti G. Barbieri F. (2006). A randomized phase II trial evaluating standard (50 mg/min) versus low (10 mg/min) infusion duration of gemcitabine as first-line treatment in advanced non-small-cell lung cancer patients who are not eligible for platinum-based chemotherapy. Lung Cancer Amst. Neth. 52, 319–325. 10.1016/j.lungcan.2006.03.004 16630670

[B19] Carrasco R. Castillo R. L. Gormaz J. G. Carrillo M. Thavendiranathan P. (2021). Role of oxidative stress in the mechanisms of anthracycline-induced cardiotoxicity: Effects of preventive strategies. Oxid. Med. Cell. Longev. 2021, 8863789. 10.1155/2021/8863789 33574985 PMC7857913

[B20] Chan S. H. Y. Khatib Y. Webley S. Layton D. Salek S. (2020). Identification of cardiotoxicity related to cancer treatments: A systematic review. PROSPERO 2020 CRD42020191760 Available from: https://www.crd.york.ac.uk/prospero/display_record.php?ID=CRD42020191760. 10.3389/fphar.2023.1137983 PMC10294714 37383708

[B21] Chang A. Y. Kim K. Glick J. Anderson T. Karp D. Johnson D. (1993). Phase II study of taxol, merbarone, and piroxantrone in stage IV non-small-cell lung cancer: The Eastern Cooperative Oncology Group Results. J. Natl. Cancer Inst. 85, 388–394. 10.1093/jnci/85.5.388 8094467

[B22] Choy H. Schwartzberg L. Dakhil S. Garon E. Gerber D. Choksi J. (2013). Phase 2 study of pemetrexed plus carboplatin, or pemetrexed plus cisplatin with concurrent radiation therapy followed by pemetrexed consolidation in patients with favorable-prognosis inoperable stage IIIA/B non-small-cell lung cancer. J. Thorac. Oncol. 8, 1308–1316. 10.1097/JTO.0b013e3182a02546 23981966 PMC4715866

[B23] Chung R. Tyebally S. Chen D. Kapil V. Walker J. M. Addison D. (2020). Hypertensive cardiotoxicity in cancer treatment—systematic analysis of adjunct, conventional chemotherapy, and novel therapies—epidemiology, incidence, and pathophysiology. J. Clin. Med. 9, 3346. 10.3390/jcm9103346 33081013 PMC7603211

[B24] Cortot A. B. Audigier-Valette C. Molinier O. Le Moulec S. Barlesi F. Zalcman G. (2020). Weekly paclitaxel plus bevacizumab versus docetaxel as second- or third-line treatment in advanced non-squamous non-small-cell lung cancer: Results of the IFCT-1103 ULTIMATE study. Eur. J. Cancer 131, 27–36. 10.1016/j.ejca.2020.02.022 32276179

[B25] Csapo M. Lazar L. (2014). Chemotherapy-induced cardiotoxicity: Pathophysiology and prevention. Med. Pharm. Rep. 87, 135–142. 10.15386/cjmed-339 PMC4508592 26528012

[B26] Curigliano G. Cardinale D. Dent S. Criscitiello C. Aseyev O. Lenihan D. (2016). Cardiotoxicity of anticancer treatments: Epidemiology, detection, and management. Ca. Cancer J. Clin. 66, 309–325. 10.3322/caac.21341 26919165

[B27] Currow D. Temel J. S. Abernethy A. Milanowski J. Friend J. Fearon K. C. (2017). Romana 3: A phase 3 safety extension study of anamorelin in advanced non-small-cell lung cancer (NSCLC) patients with cachexia. Ann. Oncol. 28, 1949–1956. 10.1093/annonc/mdx192 28472437 PMC5834076

[B28] DeSantis C. E. Lin C. C. Mariotto A. B. Siegel R. L. Stein K. D. Kramer J. L. (2014). Cancer treatment and survivorship statistics, 2014: Cancer treatment and survivorship statistics, 2014. CA. Cancer J. Clin. 64, 252–271. 10.3322/caac.21235 24890451

[B29] Dobbin S. J. H. Petrie M. C. Myles R. C. Touyz R. M. Lang N. N. (2021). Cardiotoxic effects of angiogenesis inhibitors. Clin. Sci. 135, 71–100. 10.1042/CS20200305 PMC7812690 33404052

[B30] Douillard J. Peschel C. Shepherd F. Paz-Ares L. Arnold A. Davis M. (2004). Randomized phase II feasibility study of combining the matrix metalloproteinase inhibitor BMS-275291 with paclitaxel plus carboplatin in advanced non-small cell lung cancer. Lung Cancer 46, 361–368. 10.1016/j.lungcan.2004.05.009 15541822

[B31] Eli Lilly and Company (2019a). A randomised Phase 2 trial of pemetrexed and gefitinib versus gefitinib as first line treatment for patients with stage IV non-squamous non-small cell lung cancer with activating epidermal growth factor receptor mutations (clinical trial registration No. NCT01469000). clinicaltrials.gov.

[B32] Eli Lilly and Company (2015). A randomized multicenter Phase III study of taxane/carboplatin/cetuximab versus taxane/carboplatin as first-line treatment for patients with advanced/metastatic non-small cell lung cancer (clinical trial registration No. NCT00112294). clinicaltrials.gov.

[B33] Eli Lilly and Company (2022). A randomized, multicenter, open-label Phase 3 study of gemcitabine-cisplatin chemotherapy plus necitumumab (IMC-11F8) versus gemcitabine-cisplatin chemotherapy alone in the first-line treatment of patients with stage IV squamous non-small cell lung cancer (NSCLC) (clinical trial registration No. NCT00981058). clinicaltrials.gov.

[B34] Eli Lilly and Company (2021). A randomized, multicenter, open-label Phase 3 study of pemetrexed-cisplatin chemotherapy plus necitumumab (IMC-11F8) versus pemetrexed-cisplatin chemotherapy alone in the first-line treatment of patients with stage IV nonsquamous non-small cell lung cancer (NSCLC) (clinical trial registration No. NCT00982111). clinicaltrials.gov.

[B35] Eli Lilly and Company (2019b). A randomized, multicenter, open-label, Phase 2 study of paclitaxel-carboplatin chemotherapy plus necitumumab (IMC-11F8) versus paclitaxel-carboplatin chemotherapy alone in the first-line treatment of patients with stage IV squamous non-small cell lung cancer (NSCLC) (clinical trial registration No. NCT01769391). clinicaltrials.gov.

[B36] EU Clinical Trials Register (2011). EudraCT number 2011-000634-11 - clinical trial results - EU clinical trials register WWW document. Available at: https://www.clinicaltrialsregister.eu/ctr-search/trial/2011-000634-11/results .

[B37] Ewer M. S. Ewer S. M. (2015). Cardiotoxicity of anticancer treatments. Nat. Rev. Cardiol. 12, 547–558. 10.1038/nrcardio.2015.65 25962976

[B38] Feld R. Wierzbicki R. Walde P. L. Shepherd F. A. Evans W. K. Gupta S. (1992). Phase I-II study of high-dose epirubicin in advanced non-small-cell lung cancer. J. Clin. Oncol. Off. J. Am. Soc. Clin. Oncol. 10, 297–303. 10.1200/JCO.1992.10.2.297 1310105

[B39] Ferlay J. Ervik M. Lam F. Colombet M. Mery L. Piñeros M. , (2020). Global cancer observatory: Cancer today. Lyon: International Agency for Research on Cancer.

[B40] Fukuda M. Kitazaki T. Ogawara D. Ichiki M. Mukae H. Maruyama R. (2019). Randomized phase II study of pemetrexed or pemetrexed plus bevacizumab for elderly patients with previously untreated non-squamous non-small cell lung cancer: Results of the Lung Oncology Group in Kyushu (LOGIK1201). Lung Cancer 132, 1–8. 10.1016/j.lungcan.2019.01.008 31097081

[B41] Gatzemeier U. Groth G. Butts C. Van Zandwijk N. Shepherd F. Ardizzoni A. (2004). Randomized phase II trial of gemcitabine–cisplatin with or without trastuzumab in HER2-positive non-small-cell lung cancer. Ann. Oncol. 15, 19–27. 10.1093/annonc/mdh031 14679114

[B42] GlaxoSmithKline (2014). A Phase II, open-label, multicenter, randomized study to assess the efficacy and safety of GSK1120212 compared with docetaxel in 2nd line subjects with targeted mutations (KRAS, NRAS, BRAF, MEK1) in locally advanced or metastatic non-small cell lung cancer (NSCLC stage IV) (clinical trial registration No. NCT01362296). clinicaltrials.gov.

[B43] GlaxoSmithKline (2019). Multi-arm, non-randomized, open-label Phase IB study to evaluate GSK3052230 in combination with paclitaxel and carboplatin, or docetaxel or as single agent in subjects with solid malignancies and deregulated FGF pathway signaling (clinical trial registration No. NCT01868022). clinicaltrials.gov.

[B44] Gollerkeri A. Harrold L. Rose M. Jain D. Burtness B. A. (2001). Use of paclitaxel in patients with pre-existing cardiomyopathy: A review of our experience. Int. J. Cancer 93, 139–141. 10.1002/ijc.1295 11391633

[B45] Gridelli C. Cigolari S. Gallo C. Manzione L. Ianniello G. P. Frontini L. (2001). Activity and toxicity of gemcitabine and gemcitabine +vinorelbine in advanced non-small-cell lung cancer elderly patients Phase II data from the Multicenter Italian Lung Cancer in the Elderly Study (MILES) randomized trial. Lung Cancer 8. 10.1016/s0169-5002(00)00194-x 11165408

[B46] Groen H. J. M. Sietsma H. Vincent A. Hochstenbag M. M. H. van Putten J. W. G. van den Berg A. (2011). Randomized, placebo-controlled phase III study of docetaxel plus carboplatin with celecoxib and cyclooxygenase-2 expression as a biomarker for patients with advanced non–small-cell lung cancer: The NVALT-4 study. J. Clin. Oncol. 29, 4320–4326. 10.1200/JCO.2011.35.5214 21990410

[B47] Hahn V. S. Lenihan D. J. Ky B. (2014). Cancer therapy–induced cardiotoxicity: Basic mechanisms and potential cardioprotective therapies. J. Am. Heart Assoc. 3, e000665. 10.1161/JAHA.113.000665 24755151 PMC4187516

[B48] Heigener D. F. von Pawel J. Eschbach C. Brune A. Schmittel A. Schmelter T. (2013). Prospective, multicenter, randomized, independent-group, open-label phase II study to investigate the efficacy and safety of three regimens with two doses of sagopilone as second-line therapy in patients with stage IIIB or IV non-small-cell lung cancer. Lung Cancer 80, 319–325. 10.1016/j.lungcan.2013.02.007 23522488

[B49] Herbst R. S. Ansari R. Bustin F. Flynn P. Hart L. Otterson G. A. (2011). Efficacy of bevacizumab plus erlotinib versus erlotinib alone in advanced non-small-cell lung cancer after failure of standard first-line chemotherapy (BeTa): A double-blind, placebo-controlled, phase 3 trial. Lancet 377, 1846–1854. 10.1016/S0140-6736(11)60545-X 21621716 PMC4134127

[B50] Hida T. Nokihara H. Kondo M. Kim Y. H. Azuma K. Seto T. (2017). Alectinib versus crizotinib in patients with ALK-positive non-small-cell lung cancer (J-ALEX): An open-label, randomised phase 3 trial. Lancet lond. Engl. 390, 29–39. 10.1016/S0140-6736(17)30565-2 28501140

[B51] Higgins J. Thomas J. Chandler J. Cumpston M. Li T. Page M. (2022). Cochrane Handbook for systematic reviews of Interventions. Cochrane. (updated February 2022). 10.1002/14651858.ED000142 PMC10284251 31643080

[B52] Howlader N. Ries L. A. G. Mariotto A. B. Reichman M. E. Ruhl J. Cronin K. A. (2010). Improved Estimates of cancer-specific survival rates from population-based data. JNCI J. Natl. Cancer Inst. 102, 1584–1598. 10.1093/jnci/djq366 20937991 PMC2957430

[B53] Huang M. Gong Y. Zhu J. Qin Y. Peng F. Ren L. (2020). A phase I dose-reduction study of apatinib combined with pemetrexed and carboplatin in untreated EGFR and ALK negative stage IV non-squamous NSCLC. Invest. New Drugs 38, 478–484. 10.1007/s10637-019-00811-6 31231786

[B54] IQVIA (2021). Global oncology Trends 2021.

[B55] Jänne P. A. Paz-Ares L. Oh Y. Eschbach C. Hirsh V. Enas N. (2014). Randomized, double-blind, phase II trial comparing gemcitabine-cisplatin plus the LTB4 antagonist LY293111 versus gemcitabine-cisplatin plus placebo in first-line non–small-cell lung cancer. J. Thorac. Oncol. 9, 126–131. 10.1097/JTO.0000000000000037 24346102

[B56] Jemal A. Ward E. Hao Y. Thun M. (2005). Trends in the leading causes of death in the United States, 1970-2002. JAMA 294, 1255–1259. 10.1001/jama.294.10.1255 16160134

[B57] Jemal A. Ward E. Thun M. (2010). Declining death rates reflect progress against cancer. PLoS ONE 5, e9584. 10.1371/journal.pone.0009584 20231893 PMC2834749

[B58] Jie Wang X. Miao K. Luo Y. Li R. Shou T. Wang P. (2018). Randomized controlled trial of endostar combined with cisplatin/pemetrexed chemotherapy for elderly patients with advanced malignant pleural effusion of lung adenocarcinoma. J. BUON 23, 92–97. 29552766

[B59] Johnson B. E. Kabbinavar F. Fehrenbacher L. Hainsworth J. Kasubhai S. Kressel B. (2013). Atlas: Randomized, double-blind, placebo-controlled, phase IIIB trial comparing bevacizumab therapy with or without erlotinib, after completion of chemotherapy, with bevacizumab for first-line treatment of advanced non–small-cell lung cancer. J. Clin. Oncol. 31, 3926–3934. 10.1200/JCO.2012.47.3983 24101054

[B60] Johnson D. H. Fehrenbacher L. Novotny W. F. Herbst R. S. Nemunaitis J. J. Jablons D. M. (2004). Randomized phase II trial comparing bevacizumab plus carboplatin and paclitaxel with carboplatin and paclitaxel alone in previously untreated locally advanced or metastatic non-small-cell lung cancer. J. Clin. Oncol. 22, 2184–2191. 10.1200/JCO.2004.11.022 15169807

[B61] Kato T. Seto T. Nishio M. Goto K. Yamamoto N. Okamoto I. (2018). Erlotinib plus bevacizumab phase ll study in patients with advanced non-small-cell lung cancer (JO25567): Updated safety results. Drug Saf. 41, 229–237. 10.1007/s40264-017-0596-0 29043496 PMC5808045

[B62] Kerkelä R. Grazette L. Yacobi R. Iliescu C. Patten R. Beahm C. (2006). Cardiotoxicity of the cancer therapeutic agent imatinib mesylate. Nat. Med. 12, 908–916. 10.1038/nm1446 16862153

[B63] Koch A. Bergman B. Holmberg E. Sederholm C. Ek L. Kosieradzki J. (2011). Effect of celecoxib on survival in patients with advanced non-small cell lung cancer: A double blind randomised clinical phase III trial (cyclus study) by the Swedish lung cancer study group. Eur. J. Cancer 47, 1546–1555. 10.1016/j.ejca.2011.03.035 21565487

[B64] Kosmidis P. A. Kalofonos H. P. Christodoulou C. Syrigos K. Makatsoris T. Skarlos D. (2008). Paclitaxel and gemcitabine versus carboplatin and gemcitabine in patients with advanced non-small-cell lung cancer. A phase III study of the Hellenic Cooperative Oncology Group. Ann. Oncol. 19, 115–122. 10.1093/annonc/mdm430 17938425

[B65] Kotsakis A. Kentepozidis N. Emmanouilidis Ch. Polyzos A. Agelidou A. Vaslamatzis M. (2015). Sequential administration of vinorelbine plus cisplatin and bevacizumab followed by docetaxel plus gemcitabine and bevacizumab compared to docetaxel plus cisplatin and bevacizumab regimen as first-line therapy for advanced or metastatic non-squamous non-small cell lung cancer: A multicenter randomized phase II trial of the hellenic oncology research group (horg). Lung Cancer 88, 57–62. 10.1016/j.lungcan.2015.01.012 25662596

[B66] Kubota K. Yoshioka H. Oshita F. Hida T. Yoh K. Hayashi H. (2017). Phase III, randomized, placebo-controlled, double-blind trial of motesanib (AMG-706) in combination with paclitaxel and carboplatin in east asian patients with advanced nonsquamous non–small-cell lung cancer. J. Clin. Oncol. 35, 3662–3670. 10.1200/JCO.2017.72.7297 28902534

[B67] Langer C. J. Paz-Ares L. G. Wozniak A. J. Gridelli C. de Marinis F. Pujol J.-L. (2017). Safety analyses of pemetrexed-cisplatin and pemetrexed maintenance therapies in patients with advanced non-squamous NSCLC: Retrospective analyses from 2 phase III studies. Clin. Lung Cancer 18, 489–496. 10.1016/j.cllc.2017.04.003 28479368

[B68] Lara P. N. Moon J. Hesketh P. J. Redman M. W. Williamson S. K. Akerley W. L. (2016). Swog S0709: Randomized phase II trial of erlotinib versus erlotinib plus carboplatin/paclitaxel in patients with advanced non–small cell lung cancer and impaired performance status as selected by a serum proteomics assay. J. Thorac. Oncol. 11, 420–425. 10.1016/j.jtho.2015.11.003 26725184 PMC4775366

[B69] León-Mateos L. Mosquera J. Antón Aparicio L. (2015). Treatment of sunitinib-induced hypertension in solid tumor by nitric oxide donors. Redox Biol. 6, 421–425. 10.1016/j.redox.2015.09.007 26386874 PMC4588456

[B70] Li J. Qin S. Xu J. Guo W. Xiong J. Bai Y. (2013). Apatinib for chemotherapy-refractory advanced metastatic gastric cancer: Results from a randomized, placebo-controlled, parallel-arm, phase II trial. J. Clin. Oncol. 31, 3219–3225. 10.1200/JCO.2013.48.8585 23918952

[B71] Martoni A. Guaraldi M. Piana E. (1999). Anthracyclines in non-small-cell lung cancer: Do they have a therapeutic role? Ann. Oncol. 10, S19–S23. 10.1093/annonc/10.suppl_5.S19 10582134

[B72] Martoni A. Melotti B. Guaraldi M. Pannuti F. (1991). Activity of high-dose epirubicin in advanced non-small cell lung cancer. Eur. J. Cancer 27, 1231–1234. 10.1016/0277-5379(91)90087-T 1659841

[B73] Maurea N. Coppola C. Piscopo G. Galletta F. Riccio G. Esposito E. (2016). Pathophysiology of cardiotoxicity from target therapy and angiogenesis inhibitors. J. Cardiovasc. Med. 17, S19–S26. 10.2459/JCM.0000000000000377 27183521

[B74] McGowan J. V. Chung R. Maulik A. Piotrowska I. Walker J. M. Yellon D. M. (2017). Anthracycline chemotherapy and cardiotoxicity. Cardiovasc. Drugs Ther. 31, 63–75. 10.1007/s10557-016-6711-0 28185035 PMC5346598

[B75] Mellor H. R. Bell A. R. Valentin J.-P. Roberts R. R. A. (2011). Cardiotoxicity associated with targeting kinase pathways in cancer. Toxicol. Sci. Off. J. Soc. Toxicol. 120, 14–32. 10.1093/toxsci/kfq378 21177772

[B76] Miller R. P. Tadagavadi R. K. Ramesh G. Reeves W. B. (2010). Mechanisms of cisplatin nephrotoxicity. Toxins 2, 2490–2518. 10.3390/toxins2112490 22069563 PMC3153174

[B77] Mizugaki H. Yamamoto N. Nokihara H. Fujiwara Y. Horinouchi H. Kanda S. (2015). A phase 1 study evaluating the pharmacokinetics and preliminary efficacy of veliparib (ABT-888) in combination with carboplatin/paclitaxel in Japanese subjects with non-small cell lung cancer (NSCLC). Cancer Chemother. Pharmacol. 76, 1063–1072. 10.1007/s00280-015-2876-7 26433581 PMC4612330

[B78] Moslehi J. J. (2016). Cardiovascular toxic effects of targeted cancer therapies. N. Engl. J. Med. 375, 1457–1467. 10.1056/NEJMra1100265 27732808

[B79] Movsas B. Scott C. Langer C. Werner-Wasik M. Nicolaou N. Komaki R. (2005). Randomized trial of amifostine in locally advanced non–small-cell lung cancer patients receiving chemotherapy and hyperfractionated radiation: Radiation therapy oncology group trial 98-01. J. Clin. Oncol. 23, 2145–2154. 10.1200/JCO.2005.07.167 15800308

[B80] Mudd T. W. Khalid M. Guddati A. K. (2021). Cardiotoxicity of chemotherapy and targeted agents. Am. J. Cancer Res. 11, 1132–1147. 33948350 PMC8085845

[B81] National Cancer Institute (NCI) (2019). A Phase II randomized study of OSI-774 (erlotinib) (NSC #718781) with or without carboplatin/paclitaxel in patients with previously untreated adenocarcinoma of the lung who never smoked or were former light smokers (clinical trial registration No. NCT00126581). clinicaltrials.gov.

[B82] Novello S. Scagliotti G. Sydorenko O. Vynnychenko I. Volovat C. Schneider C.-P. (2014b). Motesanib plus carboplatin/paclitaxel in patients with advanced squamous non-small-cell lung cancer results from the randomized controlled MONET1 study. J. Thorac. Oncol. 9, 1154–1161. 10.1097/JTO.0000000000000227 25157768

[B83] Novello S. Besse B. Felip E. Barlesi F. Mazieres J. Zalcman G. (2014a). A phase II randomized study evaluating the addition of iniparib to gemcitabine plus cisplatin as first-line therapy for metastatic non-small-cell lung cancer. Ann. Oncol. Off. J. Eur. Soc. Med. Oncol. 25, 2156–2162. 10.1093/annonc/mdu384 25139550

[B85] Oun R. Rowan E. (2017). Cisplatin induced arrhythmia; electrolyte imbalance or disturbance of the SA node? Eur. J. Pharmacol. 811, 125–128. 10.1016/j.ejphar.2017.05.063 28599874

[B86] Page M. J. McKenzie J. E. Bossuyt P. M. Boutron I. Hoffmann T. C. Mulrow C. D. (2021a). The PRISMA 2020 statement: An updated guideline for reporting systematic reviews. BMJ n71, n71. 10.1136/bmj.n71 PMC8005924 33782057

[B87] Page M. J. Moher D. Bossuyt P. M. Boutron I. Hoffmann T. C. Mulrow C. D. (2021b). PRISMA 2020 explanation and elaboration: Updated guidance and exemplars for reporting systematic reviews. BMJ n160, n160. 10.1136/bmj.n160 PMC8005925 33781993

[B88] Park C.-K. Oh I.-J. Kim K.-S. Choi Y.-D. Jang T.-W. Kim Y.-S. (2017). Randomized phase III study of docetaxel plus cisplatin versus pemetrexed plus cisplatin as first-line treatment of nonsquamous non-small-cell lung cancer: A trail trial. Clin. Lung Cancer 18, e289–e296. 10.1016/j.cllc.2017.01.002 28185792

[B89] Passardi A. Cecconetto L. Dall’Agata M. Dazzi C. Pasquini E. Oliverio G. (2008). Randomized phase II study with two gemcitabine- and docetaxel-based combinations as first-line chemotherapy for metastatic non-small cell lung cancer. J. Transl. Med. 6, 65. 10.1186/1479-5876-6-65 18976450 PMC2583994

[B90] Paz-Ares L. Hirsh V. Zhang L. de Marinis F. Yang J. Wakelee H. (2015). Monotherapy administration of sorafenib in patients with non-small cell lung cancer (MISSION) trial: A phase III, multicenter, placebo-controlled trial of sorafenib in patients with relapsed or refractory predominantly nonsquamous non-small-cell lung cancer after 2 or 3 previous treatment regimens. J. Thorac. Oncol. 10, 1745–1753. 10.1097/JTO.0000000000000693 26743856

[B91] Perez E. A. Rodeheffer R. (2004). Clinical cardiac tolerability of trastuzumab. J. Clin. Oncol. Off. J. Am. Soc. Clin. Oncol. 22, 322–329. 10.1200/JCO.2004.01.120 14722042

[B92] Reck M. Mellemgaard A. von Pawel J. Gottfried M. Bondarenko I. Cheng Y. (2015). Anti-angiogenic-specific adverse events in patients with non-small cell lung cancer treated with nintedanib and docetaxel. Lung Cancer 90, 267–273. 10.1016/j.lungcan.2015.08.003 26415992

[B93] Reinmuth N. Bryl M. Bondarenko I. Syrigos K. Vladimirov V. Zereu M. (2019). PF-06439535 (a bevacizumab biosimilar) compared with reference bevacizumab (Avastin®), both plus paclitaxel and carboplatin, as first-line treatment for advanced non-squamous non-small-cell lung cancer: A randomized, double-blind study. BioDrugs 33, 555–570. 10.1007/s40259-019-00363-4 31338773 PMC6790355

[B94] Saito K. Takeda K. Imanaka-Yoshida K. Imai H. Sekine T. Kamikura Y. (2003). Assessment of fatty acid metabolism in taxan-induced myocardial damage with iodine-123 BMIPP SPECT: Comparative study with myocardial perfusion, left ventricular function, and histopathological findings. Ann. Nucl. Med. 17, 481–488. 10.1007/BF03006439 14575384

[B95] Santoni M. Guerra F. Conti A. Lucarelli A. Rinaldi S. Belvederesi L. (2017). Incidence and risk of cardiotoxicity in cancer patients treated with targeted therapies. Cancer Treat. Rev. 59, 123–131. 10.1016/j.ctrv.2017.07.006 28818671

[B96] Sebastian M. Schröder A. Scheel B. Hong H. S. Muth A. von Boehmer L. (2019). A phase I/IIa study of the mRNA-based cancer immunotherapy CV9201 in patients with stage IIIB/IV non-small cell lung cancer. Cancer Immunol. Immunother. CII 68, 799–812. 10.1007/s00262-019-02315-x 30770959 PMC11028316

[B97] Sequist L. V. Yang J. C.-H. Yamamoto N. O’Byrne K. Hirsh V. Mok T. (2013). Phase III study of afatinib or cisplatin plus pemetrexed in patients with metastatic lung adenocarcinoma with *EGFR* mutations. J. Clin. Oncol. 31, 3327–3334. 10.1200/JCO.2012.44.2806 23816960

[B98] Seto T. Kato T. Nishio M. Goto K. Atagi S. Hosomi Y. (2014). Erlotinib alone or with bevacizumab as first-line therapy in patients with advanced non-squamous non-small-cell lung cancer harbouring EGFR mutations (JO25567): An open-label, randomised, multicentre, phase 2 study. Lancet Oncol. 15, 1236–1244. 10.1016/S1470-2045(14)70381-X 25175099

[B99] Shah C. Bishnoi R. Jain A. Bejjanki H. Xiong S. Wang Y. (2018). Cardiotoxicity associated with carfilzomib: Systematic review and meta-analysis. Leuk. Lymphoma 59, 2557–2569. 10.1080/10428194.2018.1437269 29465266

[B100] Shaw A. T. Kim D.-W. Nakagawa K. Seto T. Crinó L. Ahn M.-J. (2013). Crizotinib versus chemotherapy in advanced *ALK* -positive lung cancer. N. Engl. J. Med. 368, 2385–2394. 10.1056/NEJMoa1214886 23724913

[B101] Smit E. F. Berendsen H. H. Piers D. A. Smeets J. Riva A. Postmus P. E. (1992). A phase II study of high dose epirubicin in unresectable non small cell lung cancer. Br. J. Cancer 65, 405–408. 10.1038/bjc.1992.82 1313691 PMC1977612

[B102] Smith L. A. Cornelius V. R. Plummer C. J. Levitt G. Verrill M. Canney P. (2010). Cardiotoxicity of anthracycline agents for the treatment of cancer: Systematic review and meta-analysis of randomised controlled trials. BMC Cancer 10, 337. 10.1186/1471-2407-10-337 20587042 PMC2907344

[B103] Solomon B. J. Mok T. Kim D.-W. Wu Y.-L. Nakagawa K. Mekhail T. (2014). First-line crizotinib versus chemotherapy in *ALK* -positive lung cancer. N. Engl. J. Med. 371, 2167–2177. 10.1056/NEJMoa1408440 25470694

[B104] Srinivasa G. Gupta M. Seam R. Rana S. Verma S. Gupta M. (2020). A randomized prospective study comparing concomitant chemoradiotherapy using paclitaxel-carboplatin with concomitant chemoradiotherapy using etoposide-cisplatin in inoperable or nonresectable locally advanced non-small cell lung cancer. Clin. Cancer Investig. J. 9, 27. 10.4103/ccij.ccij_94_19

[B105] Stathopoulos G. P. Veslemes M. Georgatou N. Antoniou D. Giamboudakis P. Katis K. (2004). Front-line paclitaxel–vinorelbine versus paclitaxel–carboplatin in patients with advanced non-small-cell lung cancer: A randomized phase III trial. Ann. Oncol. 15, 1048–1055. 10.1093/annonc/mdh260 15205198

[B106] Swain S. M. Whaley F. S. Ewer M. S. (2003). Congestive heart failure in patients treated with doxorubicin: A retrospective analysis of three trials. Cancer 97, 2869–2879. 10.1002/cncr.11407 12767102

[B107] Tan C. Tasaka H. Yu K.-P. Murphy M. L. Karnofsky D. A. (1967). Daunomycin, an antitumor antibiotic, in the treatment of neoplastic disease.Clinical evaluation with special reference to childhood leukemia. Cancer 20, 333–353. 10.1002/1097-0142(1967)20:3<333:AID-CNCR2820200302>3.0.CO;2-K 4290058

[B108] Tocchetti C. G. Cadeddu C. Di Lisi D. Femminò S. Madonna R. Mele D. (2019). From molecular mechanisms to clinical management of antineoplastic drug-induced cardiovascular toxicity: A translational overview. Antioxid. Redox Signal. 30, 2110–2153. 10.1089/ars.2016.6930 28398124 PMC6529857

[B109] Umsawasdi T. Valdivieso M. Booser D. Barkley H. Ewer M. MacKay B. (1989). Weekly doxorubicin versus doxorubicin every 3 weeks in cyclophosphamide, doxorubicin, and cisplatin chemotherapy for non-small cell lung cancer. Cancer 64, 1995–2000. 10.1002/1097-0142(19891115)64:10<1995:aid-cncr2820641004>3.0.co;2-l 2553235

[B110] Valdivieso M. Burgess M. A. Ewer M. S. Mackay B. Wallace S. Benjamin R. S. (1984). Increased therapeutic index of weekly doxorubicin in the therapy of non-small cell lung cancer: A prospective, randomized study. J. Clin. Oncol. Off. J. Am. Soc. Clin. Oncol. 2, 207–214. 10.1200/JCO.1984.2.3.207 6321689

[B111] Wachters F. Erjavec Z. Van Putten J. Groen H. (2003). Cardiotoxicity in advanced non-small-cell lung cancer (NSCLC) patients treated with gemcitabine and either epirubicin or cisplatin as first-line treatment. Proc. Am. Soc. Clin. Oncol. 659.

[B112] Wachters F. M. Van Der Graaf W. T. A. Groen H. J. M. (2004). Cardiotoxicity in advanced non-small cell lung cancer patients treated with platinum and non-platinum based combinations as first-line treatment. Anticancer Res. 24, 2079–2083. 15274404

[B113] Weiss A. J. Manthel R. W. (1977). Experience with the use of adriamycin in combination with other anticancer agents using a weekly schedule, with particular reference to lack of cardiac toxicity. Cancer 40, 2046–2052. 10.1002/1097-0142(197711)40:5<2046:aid-cncr2820400508>3.0.co;2-5-5 336177

[B114] Weiss A. J. Metter G. E. Fletcher W. S. Wilson W. L. Grage T. B. Ramirez G. (1976). Studies on adriamycin using a weekly regimen demonstrating its clinical effectiveness and lack of cardiac toxicity. Cancer Treat. Rep. 60, 813–822. 795537

[B115] WHOCC ATC/DDD Index [WWW Document] (2022). WHOCC. Available at: https://www.whocc.no/atc_ddd_index/ .

[B116] William W. N. Zinner R. G. Karp D. D. Oh Y. W. Glisson B. S. Phan S.-C. (2007). Phase I trial of motexafin gadolinium in combination with docetaxel and cisplatin for the treatment of non-small cell lung cancer. J. Thorac. Oncol. 2, 745–750. 10.1097/JTO.0b013e31811f4719 17762342

[B117] Wils J. Utama I. Sala L. Smeets J. Riva A. (1990). Phase II study of high-dose epirubicin in non-small cell lung cancer. Eur. J. Cancer Clin. Oncol. 26, 1140–1141. 10.1016/0277-5379(90)90271-T 1963545

[B118] World Health Organization (2020). Global Health Estimates. WHO.

[B119] Wu S. Chen J. J. Kudelka A. Lu J. Zhu X. (2008). Incidence and risk of hypertension with sorafenib in patients with cancer: A systematic review and meta-analysis. Lancet Oncol. 9, 117–123. 10.1016/S1470-2045(08)70003-2 18221915

[B120] Wu Y.-L. Cheng Y. Zhou J. Lu S. Zhang Y. Zhao J. (2020). Tepotinib plus gefitinib in patients with EGFR-mutant non-small-cell lung cancer with MET overexpression or MET amplification and acquired resistance to previous EGFR inhibitor (INSIGHT study): An open-label, phase 1b/2, multicentre, randomised trial. Lancet Respir. Med. 8, 1132–1143. 10.1016/S2213-2600(20)30154-5 32479794

[B121] Wu Y.-L. Sequist L. V. Tan E.-H. Geater S. L. Orlov S. Zhang L. (2018). Afatinib as first-line treatment of older patients with EGFR mutation-positive non-small-cell lung cancer: Subgroup analyses of the LUX-lung 3, LUX-lung 6, and LUX-lung 7 trials. Clin. Lung Cancer 19, e465–e479. 10.1016/j.cllc.2018.03.009 29653820

[B122] Yoshioka H. Katakami N. Okamoto H. Iwamoto Y. Seto T. Takahashi T. (2017). A randomized, open-label, phase III trial comparing amrubicin versus docetaxel in patients with previously treated non-small-cell lung cancer. Ann. Oncol. Off. J. Eur. Soc. Med. Oncol. 28, 285–291. 10.1093/annonc/mdw621 28426104

[B123] Zaborowska-Szmit M. Krzakowski M. Kowalski D. M. Szmit S. (2020). Cardiovascular complications of systemic therapy in non-small-cell lung cancer. J. Clin. Med. 9, 1268. 10.3390/jcm9051268 32349387 PMC7287714

[B124] Zarogoulidis P. Zarogoulidis K. Sakas D. Hohenforst-Schmidt W. Tsakiridis K. Porpodis K. (2013). Docetaxel-carboplatin in combination with erlotinib and/or bevacizumab in patients with non-small cell lung cancer. OncoTargets Ther. 125, 125–134. 10.2147/OTT.S42245 PMC3589083 23467839

[B125] Zatloukal P. Petruzelka L. Zemanova M. Havel L. Janku F. Judas L. (2004). Concurrent versus sequential chemoradiotherapy with cisplatin and vinorelbine in locally advanced non-small cell lung cancer: A randomized study. Lung Cancer 46, 87–98. 10.1016/j.lungcan.2004.03.004 15364136

[B126] Zhang S. Liu X. Bawa-Khalfe T. Lu L.-S. Lyu Y. L. Liu L. F. (2012). Identification of the molecular basis of doxorubicin-induced cardiotoxicity. Nat. Med. 18, 1639–1642. 10.1038/nm.2919 23104132

[B127] Zhang X. Zhu Y. Dong S. Zhang A. Lu Y. Li Y. (2019). Role of oxidative stress in cardiotoxicity of antineoplastic drugs. Life Sci. 232, 116526. 10.1016/j.lfs.2019.06.001 31170418

[B128] Zinner R. G. Obasaju C. K. Spigel D. R. Weaver R. W. Beck J. T. Waterhouse D. M. (2015). Pronounce: Randomized, open-label, phase III study of first-line pemetrexed + carboplatin followed by maintenance pemetrexed versus paclitaxel + carboplatin + bevacizumab followed by maintenance bevacizumab in patients ith advanced nonsquamous non–small-cell lung cancer. J. Thorac. Oncol. 10, 134–142. 10.1097/JTO.0000000000000366 25371077 PMC4276572

