# Supplementary material for: Identification of cardiotoxicity related to non-small cell lung cancer (NSCLC) treatments: A systematic review
Source: Front Pharmacol. 2023 Jun 13;14:1137983. doi: 10.3389/fphar.2023.1137983 (PMC10294714; doi:10.3389/fphar.2023.1137983)
Supplement: Supplementary file 1 [file Table1.pdf]

Supplementary Table 1: Summary of treatment details and patients' characteristics of each publication.

Reference (Publication Year)	Drug Combination	Delivery / Schedule	Treatment Period	Dose	Number Randomised to Group (M/F)	Age, Median (Range)	NSCLC Stage	Smoker Status (Former/Current/Never)
Mizugaki H, 2015 (Mizugaki et al., 2015)	Carboplatin, Paclitaxel, Veliparib	All patients received veliparib (40, 80, or 120 mg) BID orally at intervals of approximately 12 h on days 1–7 of each 21-day cycle.<br><br>Carboplatin (AUC 6 mg/mL min) and paclitaxel (200 mg/m^2) were given via intravenous (IV) administration on day 3.	21-day cycle for ≤6 cycles	V-40 mg	3 (3M)	71<br>(67 – 72)	Stage IIIB: 0<br>Stage IV: 2	Former: 3<br>Current: 0<br>Never used: 0
				V-80mg	3 (3M)	56<br>(44 – 60)	Stage IIIB: 0<br>Stage IV: 3	Former: 3<br>Current: 0<br>Never used: 0
				V-120mg	6 (4M, 2F)	67<br>(47 – 73)	Stage IIIB:1;<br>Stage IV: 4;<br>Postoperative recurrence: 1	Former: 2<br>Current: 2<br>Never used: 2
Huang M, 2020 (Huang et al., 2020)	Apatinib, Carboplatin, Pemetrexed	During each cycle, pemetrexed (500 mg/m2) and carboplatin (AUC = 5) were given intravenously on days 1. The initial dose, 750 mg qd of apatinib was based on the maximum administered dose as monotherapy and the dosage reduced from 750 mg qd, 500 mg qd, 500 mg schedule 2/1, to 250 mg qd in sequence.	21-day cycle for 4 cycles<br><br>Therapeutic evaluation was assessed every six weeks.	A-750mg	3 (1M, 2F)	53.9<br>(52.4 – 64.8)	Stage IVA: 3<br>Stage IVB: 0	Former: 0<br>Current: 1<br>Never used: 2
				A-500mg	3 (2M, 1F)	63.6<br>(52.9 – 66.6)	Stage IVA: 2<br>Stage IVB: 1	Former: 2<br>Current: 0<br>Never used: 1
				A-500mg 2/1 (500 mg/day 2 weeks on 1 week off)	6 (4M, 2F)	49.8<br>(42.2 – 63.4)	Stage IVA: 3<br>Stage IVB: 3	Former: 1<br>Current: 2<br>Never used: 3
Sebastian M, 2019 (Sebastian et al., 2019)	CV9201 - generated using proprietary RNActive® Technology	The mRNAs encoding the five antigens of CV9201 were formulated separately and administered at doses of 80, 160, or 320 µg mRNA (total dose 400, 800 or 1600µg mRNA respectively). Each CV9201 component was administered individually at up to 160 µg per injection to the thighs and upper arms. The 320 µg dose was split into two injections resulting in up to 10 injections per administration visit due to technical restrictions to 200 µL per injection with a maximal concentration of 0.8 µg/µL mRNA.	During Phase I, CV9201 was administered at weeks 1, 3, 7, 11, and 15 and at weeks 1, 2, 3, 5, and 7 during Phase IIA since early tumour progressions were observed during phase I preventing the administration of all five scheduled injections.	Cohort I - 400µg	3 (3M)	75.0<br>(SD: 12.2)	Stage IIIB: 1<br>Stage IV: 2	N/A
				Cohort II - 800µg	3 (3M)	62.7<br>(SD: 8.3)	Stage IIIB: 1<br>Stage IV: 2	N/A
				Cohort III - 1600 µg	3 (1M, 2F)	62.3<br>(SD: 9.1)	Stage IIIB: 0<br>Stage IV: 3	N/A
				Phase IIA (1600µg)	37 (22M, 15F)	64.2<br>(SD: 10.1)	Stage IIIB: 5<br>Stage IV: 32	N/A

		Individual components were administered to the same body sides at each visit during phase I and to the opposite body side at different visits in phase IIA.						
**Reference (Publication Year)**	**Drug Combination**	**Delivery / Schedule**	**Treatment Period**	**Dose**	**Number Randomised to Group (M/F)**	**Age, Median (Range)**	**NSCLC Stage**	**Smoker Status (Former/Current/Never)**
Novello S, 2014 (S. Novello et al., 2014)	Cisplatin, Iniparib, Gemcitabine	Patients were randomized to gemcitabine 1250 mg/m^2 (days 1 and 8) plus cisplatin 75 mg/m^2 (day 1), with or without iniparib 5.6 mg/kg (1-h intravenous infusion, days 1, 4, 8, and 11) every 3 weeks for six cycles.	21-day cycle for 6 cycles	GC	39 (26M, 13F)	58<br>(29 – 73)	Stage I: 1<br>Stage III: 3<br>Stage IV: 35	Former/Current: 35<br>Never used: 4
				GCI	80 (64M, 16F)	59<br>(37 – 73)	Stage I: 1<br>Stage III: 2<br>Stage IV: 77	Former/Current: 71<br>Never used: 9
Cappuzzo F, 2006 (Cappuzzo et al., 2006)	Chemotherapy, Gemcitabine	At study entry, all eligible patients were randomly assigned to chemotherapy with gemcitabine 1500mg/m^2 on days 1 and 8 every 3 weeks by standard 30 min intravenous infusion<br>(arm A), or gemcitabine 10mg/m^2/min for 150 min<br>on days 1 and 8 every 3 weeks by intravenous infusion at fixed dose rate (arm B).	21-day cycle<br><br>Following completion of therapy, patients were followed every 3<br>months.	Standard (50mg/min)	56 (46M, 10F)	72<br>(55 – 81)	Stage IIIB: 19<br>Stage IV: 38	N/A
				Low (10mg/min)	61 (52M, 9F)	73<br>(54 – 81)	Stage IIIB: 20<br>Stage IV: 41	N/A
Srinivasa GY, 2020 (Srinivasa et al., 2020)	Carboplatin, Cisplatin, Etoposide, Paclitaxel	Patients were given external beam radiotherapy (EBRT) to a total dose of 60 Gy in 30 fractions at 2 Gy/fraction along with concurrent chemotherapy of the following:<br><br>Control arm: injection cisplatin 20 mg/m^2/day intravenous (iv) and injection etoposide 50 mg/m^2/day iv days 1–5 and days 29–33 of starting radiation.<br><br>Study arm: injection paclitaxel 50 mg/m^2 IV and injection carboplatin	First follow-up was done at 6 weeks. Subsequent follow-up was twice a month for the first year,<br>followed by once in 4 months for 2 years and once in 6 months after that.	Cis-Etop	18 (16M, 2F)	57<br>(45 – 65)	Stage IIIA: 10<br>Stage IIIB: 8	Smokers: 18<br>Never: 0
				Car-Pac	18 (17M, 1F)	59<br>(45 – 65)	Stage IIIA: 9<br>Stage IIIB: 9	Smokers: 18<br>Never: 0

		AUC every Monday concomitant with radiation.						
**Reference (Publication Year)**	**Drug Combination**	**Delivery / Schedule**	**Treatment Period**	**Dose**	**Number Randomised to Group (M/F)**	**Age, Median (Range)**	**NSCLC Stage**	**Smoker Status (Former/Current/Never)**
Yoshioka H, 2017 (Yoshioka et al., 2017)	Amrubicin, Docetaxel	Amrubicin hydrochloride was intravenously administered at a dose of 35 mg/m^2/day in ~20 ml of saline or 5% glucose over ~5 min. Amrubicin was administered on days 1, 2, and 3 of each 21-day cycle.<br><br>Docetaxel was intravenously administered at a dose of 60 mg/m^2/day (the approved dose in Japan), in physiological saline or 5% glucose over at least 1 h on day 1 of each course.	21-day cycle	Amrubicin	98 (66M. 32F)	N/A	Stage IIIB: 5<br>Stage IV: 81<br>Postoperative recurrence: 12	Former/Current: 72<br>Never: 26
				Docetaxel	99 (69M, 30F)	N/A	Stage IIIB: 10<br>Stage IV: 74<br>Postoperative recurrence: 15	Former/Current: 73<br>Never: 26
Johnson BE, 2013 (Johnson et al., 2013)	Bevacizumab, Erlotinib<br><br>(Chemotherapy prior to trial)	Bevacizumab (15 mg/kg) with or without erlotinib (150 mg/day) to progressive disease	21-day cycle (B) / Progressive disease (E)	Bev-Plac	373 (196M, 177F)	64<br>(23 – 83)	Stage IIIB: 37<br>Stage IV: 310<br>Recurrent: 25<br>Missing: 1	Former: 178<br>Current: 129<br>Never used: 66
				Bev-Erlo	370 (193M, 177F)	64<br>(31 – 88)	Stage IIIB: 32<br>Stage IV: 317<br>Recurrent: 21	Former: 180<br>Current: 129<br>Never used: 61
Gridelli C, 2001 (Gridelli et al., 2001)	Gemcitabine, Vinorelbine	Single-agent gemcitabine is given intravenously at the dose of 1200 mg/m^2; gemcitabine and vinorelbine are given at the doses of 1000 and 25 mg/m^2; both schedules are administered on days 1 and 8 of a 21-day cycle.	21-day cycle for ≤6 cycles	Gem	49 (42M, 7F)	74<br>(70 – 82)	Stage IIIB: 17<br>Stage IV: 32	N/A
				Gem-Vin	49 (41M, 8F)	74<br>(70 – 82)	Stage IIIB: 18<br>Stage IV: 31	N/A
Martoni A, 1991 (Martoni et al., 1991)	Epirubicin	Epirubicin (120 mg/m^2, 135 mg/m^2, 150 mg/m^2 and 165 mg/m^2 was administered by intravenous bolus (5-10 min) and repeated every 3 weeks if there was recovery from myelotoxicity(WBC ≥ 4000/µ, platelets ≥ 100,000/µl).	21-day cycle	120Epi	10	N/A	N/A	N/A
				135Epi	4	N/A	N/A	N/A
				150Epi	7	N/A	N/A	N/A
				165Epi	3	N/A	N/A	N/A

Reference (Publication Year)	Drug Combination	Delivery / Schedule	Treatment Period	Dose	Number Randomised to Group (M/F)	Age, Median (Range)	NSCLC Stage	Smoker Status (Former/Current/Never)
		Treatment was interrupted when a cumulative dose of about 900 mg/m^2 was reached.						
Wu Y, 2018 (Boehringer Ingelheim, 2018a; Sequist et al., 2013; Wu et al., 2018)	Afatinib, Cisplatin, Pemetrexed	Patients received afatinib monotherapy 40 mg film-coated tablets orally once daily.<br><br>Patients received Pemetrexed 500 mg/m^2 lyophilised powder as intravenous infusion after Cisplatin 75 mg/m^2 solution for infusion as intravenous infusion on Day 1 of each 21-day treatment course up to 6 cycles.	21-day cycle for ≤6 cycles	Afatinib	230 (83M, 147F)	60.5 (SD: 10.1)	Stage IIIB: 20<br>Stage IV: 210	Former: 70<br>Current: 5<br>Never used: 155
				Pemetrexed/ Cisplatin Chemotherapy	115 (38M, 77F)	59.9 (SD: 10.0)	Stage IIIB: 17<br>Stage IV: 98	Former: 32<br>Current: 2<br>Never used: 81
Boehringer Ingelheim, 2018 (Boehringer Ingelheim, 2018b)	Afatinib, Cisplatin, Gemcitabine	Patients received afatinib film-coated tablets 40 mg once daily (q.d.) orally with possible dose escalation to 50 mg q.d. and dose reduction to 40 mg q.d. (if applicable), 30 mg q.d., or 20 mg q.d. (according to the protocol-defined dose-escalation and dose-reduction scheme), if required.<br><br>Patients received Gemcitabine (lyophilised powder) 1000 mg/m² as an intravenous infusion over 30 minutes on day 1 and day 8, cisplatin (solution for infusion) 75 mg/m² as an intravenous infusion on Day 1 of each 21-day treatment course up to 6 cycles	21-day cycle for ≤6 cycles	Afatinib	242 (87M, 155F)	56.7 (SD: 11.2)	Stage IIIB: 16<br>Stage IV: 226	Former: 44<br>Current: 17<br>Never used: 181
				Cisplatin, Gemcitabine Chemotherapy	122 (39M, 83F)	55.6 (SD: 10.1)	Stage IIIB: 6<br>Stage IV: 116	Former: 13<br>Current: 10<br>Never used: 99
Boehringer Ingelheim, 2020 (Boehringer Ingelheim, 2020)	Afatinib, Gefitinib	Afatinib film-coated tablets administered orally, once daily. Starting dose was 40 milligram (mg), dose escalation to 50mg was allowed after completing one 28-day treatment course, dose reduction to 40mg, 30mg	Until progressive disease, unacceptable toxicity, death, or a desire to withdraw.	Afatinib	160 (69M, 91F)	61.7 (SD: 11.5)	Stage IIIB: 8<br>Stage IV: 152	Former: 106<br>Current: 6<br>Never used: 181
				Gefitinib	159 (53M, 106F)	63.0 (SD: 10.4)	Stage IIIB: 3<br>Stage IV: 156	Former: 50<br>Current: 3

		or 20mg was required in the presence of protocol-defined adverse events.<br><br>Gefitinib film-coated tablets, administered orally, once daily. Starting dose was 250mg, the investigator was allowed to modify dosing in the presence of drug-related adverse events.						Never used: 106
**Reference (Publication Year)**	**Drug Combination**	**Delivery / Schedule**	**Treatment Period**	**Dose**	**Number Randomised to Group (M/F)**	**Age, Median (Range)**	**NSCLC Stage**	**Smoker Status (Former/Current/Never)**
Hida T, 2017 (Hida et al., 2017)	Alectinib, Crizotinib	Patients received oral alectinib (eight capsules totalling 300 mg; or crizotinib (one capsule totalling 250 mg; twice daily until progressive disease (unless continuation of treatment was considered clinically meaningful by a physician), unacceptable toxicity, death, or a desire to withdraw.	Until progressive disease, unacceptable toxicity, death, or a desire to withdraw.	Alectinib	103 (41M, 62F)	61<br>(27 – 85)	Stage IIIB: 3<br>Stage IV: 76<br>Postoperative recurrence: 24	Former: 45<br>Current: 2<br>Never used: 56
				Crizotinib	104 (41M, 63F)	59.5<br>(25 – 84)	Stage IIIB: 3<br>Stage IV: 75<br>Postoperative recurrence: 26	Former: 40<br>Current: 3<br>Never used: 61
Berghmans T, 2013 (Berghmans et al., 2013)	Cisplatin, Docetaxel, Gemcitabine, Ifosfamide	Patients were randomised on a 1:1:1 ratio between IG (ifosfamide 3 g/m^2 day 1; gemcitabine $1g/m^2$ days 1+8), GIP (gemcitabine 1 g/m^2 days 1+8; ifosfamide 3 g/m^2 day 1; cisplatin 50 mg/m^2 day 1), and DP (docetaxel 75 mg/m^2 plus cisplatin 50 mg/m^2, both on day 1)	21-day cycle	IG	229 (172M, 57F)	59<br>(30 – 84)	Stage IIB: 3<br>Stage IIIA: 12<br>Stage IIIB: 27<br>Stage IV: 186<br>Missing: 1	N/A
				GIP	231 (174M, 57F)	58<br>(29 – 78)	Stage IIB: 2<br>Stage IIIA: 7<br>Stage IIIB: 30<br>Stage IV: 191<br>Missing: 1	N/A
				DP	233 (177M, 56F)	58<br>(28 – 81)	Stage IIB: 1<br>Stage IIIA: 14<br>Stage IIIB: 31<br>Stage IV: 187<br>Missing: 0	N/A
Yang JJ, 2014 & GSK, 2014 ("EudraCT Number	Docetaxel, GSK1120212 (Trametinib)	Patients were randomly assigned in a 2 : 1 ratio to trametinib 2 mg orally once daily or docetaxel 75 mg/m^2 IV every 3 weeks.	Until progressive disease, unacceptable toxicity, death, or a desire to withdraw.	Doc	43<br>(23M, 20F)	63<br>(34 – 79)	Stage IV: 43	Former: 23<br>Current: 13<br>Never used: 7
				Tra	86<br>(46M, 40F)	63<br>(40 – 79)	Stage IV: 86	Former: 67<br>Current: 13

2011-000634-11 - Clinical trial results - EU Clinical Trials Register," n.d.; GlaxoSmith Kline, 2014)								Never used: 6
**Reference (Publication Year)**	**Drug Combination**	**Delivery / Schedule**	**Treatment Period**	**Dose**	**Number Randomised to Group (M/F)**	**Age, Median (Range)**	**NSCLC Stage**	**Smoker Status (Former/Current/Never)**
Martoni A, 1999 (Martoni et al., 1999)	Epirubicin, Cisplatin, Vinorelbine	Epirubicin was administered at the dose of 120 mg/m^2 by i.v. bolus and cisplatin at the dose of 60 mg/m^2 in one hour by i.v. on day 1.<br><br>VNR was administered at the dose of 25 mg/m^2 by i.v. bolus on day 1 and 8 and<br>cisplatin as above on day 1.	21-day cycle for ≤12 cycles	HDEpi-Cis	112	Only summarised as overall<br><br>61 (42 – 72)	Only summarised as overall<br><br>Stage IIIA: 25<br>Stage IIIB: 85<br>Stage IV: 88<br>Recurrence: 14<br>Missing: 16	N/A
				Vin-Cis	116			N/A
Reck M, 2015 (Reck et al., 2015)	Docetaxel, Nintedanib	Standard intravenous docetaxel (75 mg/m^2) on day 1 and nintedanib (200 mg bid) or matching placebo on days 2–21	N/A	Doc-Nin	655 (476M, 179F)	59.7<br>(S.D. = 9.7)	Stage <IIIB: 105<br>Stage IIIB: 148<br>Stage IV: 399<br>Missing: 3	Former/Current: 490<br>Never: 165
				Doc-Plac	659 (479M, 180F)	59.8<br>(S.D. = 9.0)	Stage <IIIB: 105<br>Stage IIIB: 146<br>Stage IV: 408<br>Missing: 0	Former/Current: 498<br>Never: 161
Saito K, 2003 (Saito et al., 2003)	Carboplatin, Docetaxel, Paclitaxel	In the docetaxel group, 16 patients were treated with 60 mg/m^2 of docetaxel combined with 300 mg/m^2 of carboplatin intravenously every 3 weeks for three cycles.	21-day cycle for 3 cycles	Car-Doc	16 (10M, 6F)	60.75	N/A	N/A
				Car-Pac	9 (6M, 3F)	63.6	N/A	N/A

		In the paclitaxel group, 9 patients were treated with 180 mg/m^2 of paclitaxel combined with 300 mg/m^2 of carboplatin intravenously every 3 weeks for three cycles.						
**Reference (Publication Year)**	**Drug Combination**	**Delivery / Schedule**	**Treatment Period**	**Dose**	**Number Randomised to Group (M/F)**	**Age, Median (Range)**	**NSCLC Stage**	**Smoker Status (Former/Current/Never)**
Barlesi F, 2018 (Barlesi et al., 2018)	Avelumab, Docetaxel	Participants in the avelumab group received 10 mg/kg avelumab intravenously over 1h once every 2 weeks. An antihistamine and paracetamol (eg, oral diphenhydramine 25–50 mg and oral or intravenous paracetamol 500–600 mg, or equivalent) were given 30–60 min before each infusion of avelumab. Participants in the docetaxel group received 75 mg/m² docetaxel intravenously over 1h every 3 weeks according to label instructions and local guidelines. Dexamethasone was given before each infusion of docetaxel according to local standard of care.	Until progressive disease, unacceptable toxicity, death, or a desire to withdraw.	Ave	396 (269M, 127F)	64 (58 – 69)	N/A	Former/Current: 324<br>Never: 70<br>Missing: 2
				Doc	396 (273M, 123F)	63 (57 – 69)	N/A	Former/Current: 333<br>Never: 63
Camidge DR, 2018 (Camidge et al., 2018)	Brigatinib, Crizotinib	They were randomly assigned (in a 1:1 ratio) to receive oral brigatinib at a dose of 180 mg once daily after a 7-day lead-in period of 90 mg once daily or oral crizotinib at a dose of 250 mg twice daily.	N/A	Brig	137 (68M, 69F)	58 (27 – 86)	Stage IIIB: 8<br>Stage IV: 129	Former: 49<br>Current: 4<br>Never used: 84
			N/A	Crizo	138 (57M, 81F)	60 (29 – 89)	Stage IIIB: 12<br>Stage IV: 126	Former: 56<br>Current: 7<br>Never used: 75
Wachters FM, 2004 (Wachters et al., 2003)	Cisplatin, Epirubicin, Gemcitabine	Eligible patients were randomised to receive either cisplatin or epirubicin both with gemcitabine (1125 mg/m^2) administered during a 30-minute infusion on days 1 (before cisplatin or	21-day cycle for ≤5 cycles or until progressive disease, unacceptable toxicity, death, or a desire to withdraw.	Gem-Cis	31 (25M, 6F)	61 (45 – 76)	Stage IIIA: 2<br>Stage IIIB: 7<br>Stage IV: 22	N/A
				Gem-Epi	38 (24M, 14F)	61 (43 – 76)	Stage IIIA: 5<br>Stage IIIB: 14<br>Stage IV: 19	N/A

		epirubicin) and 8. Cisplatin 80 mg/m^2 was administered intravenously over 3 hours after prehydration with 0.9% NaCl on day 2 of each 21-day treatment cycle. Epirubicin 100 mg/m^2 was administered as an intravenous bolus injection within 5 minutes on day 1 of each 21-day cycle.						
**Reference (Publication Year)**	**Drug Combination**	**Delivery / Schedule**	**Treatment Period**	**Dose**	**Number Randomised to Group (M/F)**	**Age, Median (Range)**	**NSCLC Stage**	**Smoker Status (Former/Current/Never)**
Shaw AT, 2013 (Shaw et al., 2013)	Crizotinib, Docetaxel, Pemetrexed	Crizotinib (PF-02341066) 250 mg (administered as two 100-mg tablets and one 50-mg tablet) orally twice daily continuously in 21-day cycles.<br><br>Pemetrexed 500 mg/m^2 intravenous infusion over 10 minutes or docetaxel 75 mg/m^2 intravenous infusion over 1 hour on Day 1 of 21-day cycle.	21-day cycle, until progressive disease, unacceptable toxicity, death, or a desire to withdraw.	Criz	173 (75M, 98F)	50.3 (SD: 13.1)	N/A	Former: 59<br>Current: 5<br>Never used: 108
				Doc-Pem	174 (79M, 95F)	49.8 (SD: 13.0)	N/A	Former: 354<br>Current: 9<br>Never used: 111
Solomon BJ, 2014 (Solomon et al., 2014)	Crizotinib, Carboplatin, Cisplatin, Pemetrexed	Crizotinib 250 mg capsule, orally twice daily was administered in treatment cycle of 21 days.<br><br>Chemotherapy were administered intravenously on Day 1 of each cycle. Pemetrexed 500 mg/m^2 IV infusion according to standard of care was administered over 10 min; either cisplatin 75 mg/m^2 IV infusion was administered approximately 30 min after the end of the pemetrexed infusion or carboplatin was administered at a dose calculated to produce an AUC of 5 or 6 mg*min/mL, approximately 30 min after end of pemetrexed infusion.	21-day cycle for ≤6 cycles	Criz	172 (68M, 104F)	50.94 (SD: 11.9)	N/A	Former: 56<br>Current: 10<br>Never used: 106
				Pem-Car/Cis	171 (63M, 108F)	52.89 (SD: 13.1)	N/A	Former: 54<br>Current: 5<br>Never used: 112
Bonomi P, 2000	Cisplain, Etoposide,	Patients were randomised to one of three chemotherapy regiments:	21-day cycle, until progressive disease,	Cis-Etop	193 (127M, 66F)	61.7	Stage IIIB: 29<br>Stage IV: 164	N/A

(Bonomi et al., 2000)	Paclitaxel	(1) etoposide/cisplatin (EC) regimen: cisplatin 75mg/m^2 intravenously (IV) over 1 hour on day 1 plus etopside 100mg/m^2 IV over 45 minutes on days 1, 2 and 3;<br>(2) high dose paclitaxel (PCG) regimen: cisplatin 75 mg/m^2 IV over 1 hour on day 2 preceded by paclitaxel 250 mg/m^2 IV as a 24-hour infusion on day 1, plus filgrastim 5 mcg/kg subcutaneously beginning on day 3 and continuing until the granulocyte count was ≥ 10,000/μL;<br>(3) low dose paclitaxel (PC) regimen: cisplatin 75 mg/m^2 IV over 1 hour on day 2 preceded by paclitaxel 135 mg/m^2 IV as a 24-hour infusion starting on day 1.	unacceptable toxicity, death, or a desire to withdraw.	Cis-250Pac	191 (120M, 71F)	60.8	Stage IIIB: 38<br>Stage IV: 153	N/A
				Cis-135Pac	190 (118M, 72F)	62.7	Stage IIIB: 44<br>Stage IV: 146	N/A
**Reference (Publication Year)**	**Drug Combination**	**Delivery / Schedule**	**Treatment Period**	**Dose**	**Number Randomised to Group (M/F)**	**Age, Median (Range)**	**NSCLC Stage**	**Smoker Status (Former/Current/Never)**
Zatloukal P, 2004 (Zatloukal et al., 2004)	Cisplatin, Vinorelbine	Both arms received the same chemotherapy (CT) and radiotherapy (RT) treatment dosage, but received either concurrent treatment or sequential treatment.<br>The chemotherapy regimen consisted of a combination of cisplatin 80mg/m^2 on day 1, and vinorelbine 25mg/m^2 on day 1, 8, and 15. The dose of vinorelbine was reduced to 12.5mg/m^2 during cycles 2 and 3.	28-day cycle for ≤4 cycles	Con	52 (33M, 19F)*	62<br>(42 – 75)	Stage IIIA: 8<br>Stage IIIB: 44	N/A
				Seq	50 (36M, 14F)*	61<br>(49 – 75)	Stage IIIA: 7<br>Stage IIIB: 43	N/A
					* Three patients were not evaluated for response because of consent withdrawal in one patient before treatment start and early death during first cycle due to pulmonary embolism in two patients.			
Zarogoulidis P, 2013 (Zarogoulidis et al., 2013)	Bevacizumab, Carboplatin, Docetaxel, Erlotinib	All patients initially received two cycles of chemotherapy with docetaxel 100 mg/m^2 and carboplatin at a dose of area under the concentration-time curve of 5.5 every 28 days.<br><br>The first group (controls) received a further four cycles of docetaxel-	Each group is slightly different	Car-Doc	61 (52M, 4F, Missing: 5)	65	Stage IIIB: 10<br>Stage IV: 51	Former: 39<br>Current: 14<br>Never used: 8
				CDE	52 (40M, 12F)	62.5	Stage IIIB: 13<br>Stage IV: 39	Former: 39<br>Current: 5<br>Never used: 8
				BCD	56 (45M, 11F)	62.5	Stage IIIB: 15<br>Stage IV: 41	Former: 45<br>Current: 2<br>Never used: 9

		carboplatin and continued with observation until disease progression.<br><br>The second group (erlotinib) received four cycles of docetaxel-carboplatin plus erlotinib administered orally at 150 mg/dL per day beginning on the first day of the third cycle and continued with erlotinib monotherapy thereafter until progression.<br><br>The third group (bevacizumab) received four cycles of docetaxel-carboplatin plus bevacizumab 7.5 mg/kg by intravenous infusion every 28 days and continued with bevacizumab every 21 days until disease progression.<br><br>The fourth group (combination therapy)<br>received four cycles of chemotherapy plus bevacizumab 7.5 mg/kg every 28 days and erlotinib 150 mg/dL, and continued with bevacizumab every 21 days and erlotinib<br>until disease progression.		BCDE	60 (50M, 10F)	60	Stage IIIB: 10<br>Stage IV: 50	Former: 53<br>Current: 5<br>Never used: 2
**Reference (Publication Year)**	**Drug Combination**	**Delivery / Schedule**	**Treatment Period**	**Dose**	**Number Randomised to Group (M/F)**	**Age, Median (Range)**	**NSCLC Stage**	**Smoker Status (Former/Current/Never)**
Koch A, 2011 (Koch et al., 2011)	Celecoxib, Chemotherapy (carboplatin/cisplatin / gemcitabine / vinorelbine)	Patients were randomised to celecoxib 400 mg b.i.d. or placebo. Celecoxib or matching placebo capsules were taken from the first day of chemotherapy for a maximum of 1 year.<br><br>Chemotherapy with four cycles of a combination of a<br>platinum compound (carboplatin or cisplatin) and a third generation drug	Chemotherapy for 4 cycles, Celecoxib maximum 1 year	Celeco	158 (73M, 85F)	66<br>(38 – 85)	Stage IIIB: 37<br>Stage IV: 121	Former: 77<br>Current: 65<br>Never used: 16
				Placebo	158 (87M, 71F)	65<br>(37 – 85)	Stage IIIB: 38<br>Stage IV: 120	Former: 84<br>Current: 58<br>Never used: 16

		(primarily gemcitabine or vinorelbine) was recommended.						
**Reference (Publication Year)**	**Drug Combination**	**Delivery / Schedule**	**Treatment Period**	**Dose**	**Number Randomised to Group (M/F)**	**Age, Median (Range)**	**NSCLC Stage**	**Smoker Status (Former/Current/Never)**
Bi N, 2019 (Bi et al., 2019)	Celecoxib, Cisplatin, Etoposide	The CCRT regimen was identical in both arms. Patients were randomly assigned to CCRT alone or CCRT with celecoxib.<br>In the concurrent schedule, chemotherapy consisted of 50mg/m^2 of etoposide on days 1 to 5 and 50mg/m^2 of cisplatin on days 1 and 8, every 4 weeks (EP regimen) for 2 cycles. Celecoxib, at 200mg twice daily, was started 1 week before the initiation of radiotherapy and was continued without interruption until the end of radiotherapy.	28-day cycle for 2 cycles	CE	51 (39M, 12F)		Stage IIIA: 20<br>Stage IIIB: 31	Former/Current: 39<br>Never: 12
				CE-Cele	45 (36M, 9F)		Stage IIIA: 15<br>Stage IIIB: 30	Former/Current: 34<br>Never: 11
Herbst RS, 2011 (Herbst et al., 2011)	Bevacizumab, Erlotinib	Patients received placebo or bevacizumab administered at 15 mg/kg by intravenous infusion on the first day of 3-week cycles (±4 days). Erlotinib was taken orally at 150 mg per day, beginning on the first day of the first cycle.	21-day cycle, until progressive disease, unacceptable toxicity, death, or a desire to withdraw.	Erlo	317 (170M, 147F)	65<br>(S.D. = 10.3)	N/A	Former: 212<br>Current: 72<br>Never used: 33
				Erlo-Bev	319 (171M, 148F)	64.8<br>(S.D. = 10.4)	N/A	Former: 237<br>Current: 48<br>Never used: 34
Seto T, 2014 & Kato T, 2018 (Kato et al., 2018; Seto et al., 2014)	Bevacizumab, Erlotinib	Patients assigned to the erlotinib plus bevacizumab group received bevacizumab 15 mg/kg by intravenous infusion on day 1 of a 21-day cycle and erlotinib orally once daily at 150 mg/day, starting from day 1 of cycle 1.<br><br>Patients in the erlotinib alone group received erlotinib orally once a day at 150 mg/day.	21-day cycle, until progressive disease, unacceptable toxicity, death, or a desire to withdraw.	Erlo	77 (26M, 51F)	67.0<br>(60 – 73)	Stage IIIB: 0<br>Stage IV: 62<br>Postoperative recurrence: 15	Former: 6<br>Current: 26<br>Never used: 45
				Erlo-Bev	75 (30M, 45F)	67.0<br>(59 – 73)	Stage IIIB: 1<br>Stage IV: 60<br>Postoperative recurrence: 14	Former: 9<br>Current: 24<br>Never used: 42
				#Study 171 & 174 are duplicates, patients' characteristics from 171 and cardiotoxicity data from 174				
National Cancer	Carboplatin,	Arm A: Patients receive oral erlotinib once daily on days 1-21.	21-day cycle for ≤6 cycles or until	Erlo	81 (32M, 49F)	58<br>(32 – 78)	N/A	Former/Current: 17

Institute, 2019 (National Cancer Institute (NCI), 2019)	Erlotinib, Paclitaxel	Arm B: Apart from receiving erlotinib, patients also receive paclitaxel IV over 1-3 hours and carboplatin IV over 15-30 minutes on day 1.	progressive disease, unacceptable toxicity, death, or a desire to withdraw.<br><br>After completion of 6 courses of treatment, patients may continue to receive erlotinib alone as above.					Never: 64
				Erlo-Car-Pac	100 (42M, 58F)	60<br>(34 – 81)	N/A	Former/Current: 21<br>Never: 79

**Reference (Publication Year)**	**Drug Combination**	**Delivery / Schedule**	**Treatment Period**	**Dose**	**Number Randomised to Group (M/F)**	**Age, Median (Range)**	**NSCLC Stage**	**Smoker Status (Former/Current/Never)**
Stathopoulos GP, 2004 (Stathopoulos et al., 2004)	Carboplatin, Paclitaxel, Vinorelbine	Arm A patients received carboplatin and paclitaxel.<br>The doses were standard for carboplatin, 6 AUC (area under the curve), and for paclitaxel, 175 mg/m^2, repeated every 21 days. Paclitaxel was initially infused for 3 h followed by carboplatin at the 1-day outpatient clinic. Premedication included ondasentron 8mg IV, dexamethasone 8mg IV and diphenhydramine 50mg IV with modified timing 1 h before the beginning of treatment and repeated 4 and 8 h thereafter.<br><br>Arm B patients received paclitaxel and vinorelbine at the following doses: paclitaxel 135 mg/m^2 and vinorelbine 25 mg/m^2 repeated every 2 weeks. The<br>dose reduction of paclitaxel was counterbalanced by the earlier (once every 2 weeks instead of every 3 weeks) administration.	21-day cycle<br><br>Pac-Car: for 6 cycles<br><br>Pac-Vin: for 9 cycles	Pac-Car	185 (160M, 25F)	65<br>(30 – 83)	Stage IIIA: 15<br>Stage IIIB: 79<br>Stage IV: 91	N/A
				Pac-Vin	175 (152M, 23F)	65<br>(36 – 84)	Stage IIIA: 11<br>Stage IIIB: 81<br>Stage IV: 83	N/A
Valdivieso M, 1984	Cisplatin, Cyclo-phosphamide,	All patients received 3 g/m^2 ftorafur on days 1 and 4, 500 mg/m^2 cyclophosphamide on day 1, and 15	21-day cycle	Weekly Dox	52 (38M, 14F)	54 (36 – 78)	N/A	N/A
				Standard Dox	48 (41M, 7F)	59 (33 – 76)	N/A	N/A

Reference (Publication Year)	Drug Combination	Delivery / Schedule	Treatment Period	Dose	Number Randomised to Group (M/F)	Age, Median (Range)	NSCLC Stage	Smoker Status (Former/Current/Never)
(Valdivieso et al., 1984)	Doxorubicin, Ftorafur	mg/m^2 cisplatin IV on days 1 through 4.<br><br>Patients receiving standard doxorubicin, received 60 mg/m^2 IV on day 1 every 3 weeks. Patients receiving weekly doxorubicin received 20 mg/m^2 IV weekly.						
Baggstrom MQ, 2017 (Baggstrom et al., 2017a)	Chemotherapy, Sunitinib	Patients were registered 3 to 5 weeks after day 1 of cycle 4 of prior chemotherapy and then randomly assigned 1:1 in a double-blind fashion to receive maintenance sunitinib, 37.5 mg/d continuously, or placebo until disease progression or intolerable toxicity.	Until progressive disease, unacceptable toxicity, death, or a desire to withdraw.	CT-Placebo<br><br>(prior use of bev – Y: 24; N: 80)	104 (60M, 44F)	67.0<br>(44.0 – 89.0)<br><br>[Mean, S.D. = 66.3 ± 9.3]	Stage IIIB: 12<br>Stage IV: 92	Former: 67<br>Current: 27<br>Never used: 10
				CT-Sunitinib<br><br>(prior use of bev – Y: 23; N: 83)	106 (57M, 49F)	65.0<br>(25.0 – 84.0)<br><br>[Mean, S.D. = 63.6±10.0]	Stage IIIB: 14<br>Stage IV: 92	Former: 76<br>Current: 25<br>Never used: 5
Paz-Ares L, 2015 (Paz-Ares et al., 2015)	Best supportive care, Sorafenib	Patients were randomized 1:1 in a double-blind fashion to receive oral sorafenib (two tablets of 200 mg) plus BSC or matching placebo twice daily (morning and evening) plus BSC on a continuous basis.	Until progressive disease, unacceptable toxicity, death, or a desire to withdraw.	BSC-Placebo	353 (209M, 144F)	62.0	N/A	Former/Current: 216<br>Never: 134<br>Missing: 3
				BSC-Sorafenib	350 (186M, 164F)	59.0	N/A	Former/Current: 181<br>Never: 161<br>Missing: 8
Novello S, 2014 (S Novello et al., 2014)	Carboplatin, Paclitaxel, Motesanib	Patients in Arm A received a median of four cycles of carboplatin and paclitaxel; Patients in Arm B received a median of six cycles of carboplatin and paclitaxel.<br><br>The median daily doses of motesanib or placebo administered were 125 mg in both treatment arms.	Until progressive disease, unacceptable toxicity, death, or a desire to withdraw.	Car-Pac-Placebo	178 (150M, 28F)	59.5<br>(32 – 81)	Stage IIIB: 25<br>Stage IV / recurrent: 153	Former/Current: 159<br>Never: 19
				Car-Pac-Mote	182 (145M, 37F)	62.0<br>(31 – 79)	Stage IIIB: 28<br>Stage IV / recurrent: 154	Former/Current: 153<br>Never: 29
Akamatsu H, 2018	Carboplatin, Cisplatin,		Until progressive disease,	Osim	279 (107M, 172F)	62<br>(25 – 85)	N/A	Former: 76<br>Current: 14

(Akamatsu et al., 2018)	Pemetrexed, Osimertinib	In the osimertinib group, patients received oral osimertinib at a dose of 80 mg once daily.<br><br>In the platinum-pemetrexed group, patients received pemetrexed (IV) 500 mg/m^2 plus either carboplatin at an area under the plasma concentration-time curve of 5, or cisplatin 75 mg/every 3 weeks for up to 6 cycles.	unacceptable toxicity, death, or a desire to withdraw.					Never used: 189
				Plat (car/cis)-Pem	140 (43M, 97F)	63 (20 – 90)	N/A	Former: 38<br>Current: 8<br>Never used: 94
**Reference (Publication Year)**	**Drug Combination**	**Delivery / Schedule**	**Treatment Period**	**Dose**	**Number Randomised to Group (M/F)**	**Age, Median (Range)**	**NSCLC Stage**	**Smoker Status (Former/Current/Never)**
Kosmidis PA, 2008 (Kosmidis et al., 2008)	Carboplatin, Paclitaxel, Gemcitabine	Eligible patients received either paclitaxel 200 mg/m^2 as a 3-h infusion on day 1 in combination with a 30-min infusion of gemcitabine 1 g/m^2 on days 1 and 8 (group A) or carboplatin at an AUC of 6 mg according to the Calvert formula, as a 1-h IV infusion, in combination with a 30-min infusion of gemcitabine 1 g/m^2 on days 1 and 8 (group B).	21-day cycle, until progressive disease, unacceptable toxicity, death, or a desire to withdraw.	Gem-Pac	225 (194M, 31F)	63 (42 – 82)	Stage IIIB: 30<br>Stage IV: 195	N/A
				Gem-Car	227 (184M, 43F)	63 (36 – 83)	Stage IIIB: 31<br>Stage IV: 196	N/A
					* Only 219 patients from each group were analysed, but cardiotoxicity data of 7 patients from Group A (Gem-Pac), and 9 patients from Group B (Gem-Car) were missing.			
Reinmuth N, 2019 (Reinmuth et al., 2019)	Bevacizumab, Carboplatin, Paclitaxel, PF-06439535	On treatment days when PF-06439535 or bevacizumab-<br>was administered in combination with CT, the order of administration was paclitaxel, carboplatin, and PF-06439535 or bevacizumab. Paclitaxel was administered at an initial dose of 200 mg/m^2, carboplatin at an initial dose targeting an area under the concentration versus time curve of 6.0 mg/mL·min, and PF-06439535 or bevacizumab at an initial dose of 15 mg/kg.	21-day cycle,<br><br>After CT was dis-continued, PF-06439535<br>or bev monotherapy could be administered until progressive disease, unacceptable toxicity, death, or a desire to withdraw.	Car-Pac-Bev	361 (230M, 131F)	61.0 (31 – 83)	Stage IIIB: 29<br>Stage IV: 282<br>Recurrent: 50	Former: 135<br>Current: 117<br>Never used: 109
				Car-Pac-PF06439535	358 (237M, 121F)	62.0 (25 – 87)	Stage IIIB: 48<br>Stage IV: 265<br>Recurrent: 45	Former: 135<br>Current: 117<br>Never used: 109
Blumenschein GR, 2010 (Blumenschein et al., 2010)	Carboplatin, Paclitaxel, Panitumumab, Motesanib	In arm A, patients received motesanib orally at escalating doses of 50 mg once daily, 125 mg once daily, or 75 mg twice daily starting on day 3 of cycle 1, and then from day 1 of each	21-day cycle, for ≤6 cycles<br><br>Patients received motesanib alone	Mote(E)-CP	23 (16M, 7F)	64 (39 – 79)	Stage IIIB: 4<br>Stage IV: 19	Former: 16<br>Current: 3<br>Never used: 4
				Mote(E)-Pani	16 (10M, 6F)	58 (32 – 76)	Stage IIIB: 3<br>Stage IV: 13	Former: 9<br>Current: 3

		subsequent cycle. Paclitaxel was given concomitantly through a 3-h IV infusion at 200 mg/m^2 and carboplatin was given through a 30-min IV infusion at an area under the AUC of 6 mg/mL·min. Carboplatin/paclitaxel was administered once on day 1 of each 21-d cycle for up to six cycles.<br><br>In arm B, patients received motesanib orally beginning on day 3 at escalating doses at 50 mg once daily, 125 mg once daily, or 75 mg twice daily plus panitumumab 9 mg/kg through IV infusion on day 1 of each 21-d cycle.<br><br>In arm C, patients received 125 mg once daily motesanib plus carboplatin/paclitaxel beginning on day 3 (dose and schedule as described in arm A) and panitumumab (9 mg/kg through IV infusion on day 1 of each 21-d cycle). Enrollment in arm C started after the 125 mg once daily dose had been established as safe and tolerable in treatment arms A and B. There was no dose escalation in arm C. Car/Pac treatment and panitumumab dosing followed the same rules as outlined for arms A and B, respectively.	until progressive disease, unacceptable toxicity, death, or a desire to withdraw.					Never used: 4
				Mote(125)-CP-Pani	6 (3M, 3F)	60<br>(56 – 64)	Stage IIIB: 1<br>Stage IV: 5	Former: 6<br>Current: 0<br>Never used: 0
**Reference (Publication Year)**	**Drug Combination**	**Delivery / Schedule**	**Treatment Period**	**Dose**	**Number Randomised to Group (M/F)**	**Age, Median (Range)**	**NSCLC Stage**	**Smoker Status (Former/Current/Never)**
Choy H, 2013 (Choy et al., 2013)	Carboplatin, Cisplatin, Pemetrexed	Arm A: pemetrexed 500 mg/m^2 (IV) administered on day 1 followed by carboplatin area under the curve five intravenously on day 1.	21-day cycle, for 3 cycles	Pem-Car	46 (30M, 16F)	62.8<br>(43.7 – 82.4)	Stage IIIA: 20<br>Stage IIIB: 26	N/A
				Pem-Cis	52 (31M, 21F)	64.3<br>(45.8 – 85.2)	Stage IIIA: 27<br>Stage IIIB: 25	N/A

		Arm B: pemetrexed 500 mg/m^2 (IV) administered on day 1 followed by cisplatin 75 mg/m2 on day 1.						
**Reference (Publication Year)**	**Drug Combination**	**Delivery / Schedule**	**Treatment Period**	**Dose**	**Number Randomised to Group (M/F)**	**Age, Median (Range)**	**NSCLC Stage**	**Smoker Status (Former/Current/Never)**
William WN, 2007 (William et al., 2007)	Cisplatin, Docetaxel, Motexafin gadolinium	For all cohorts, docetaxel 75 mg/m^2 and cisplatin 75 mg/m^2 were given on day 1 plus different doses of MGd for each cohort.<br><br>Cohort 1, MGd 2.5 mg/kg on day 1<br>Cohort 2, MGd 5 mg/kg on day 1<br>Cohort 3, MGd 10 mg/kg on day 1<br>Cohort 4, MGd 7.5 mg/kg on days 1 and 2		2.5MGd	3	Only have overall data	Only have overall data	Only have overall data
				5MGd	6			
				10MGd	7			
				15MGd	5			
Chang AY, 1993 (Chang et al., 1993)	Merbarone, Piroxantrone, Taxol	Merbarone – 1000 mg/m^2 merbarone by continuous intravenous infusion through a central catheter daily for 5 days.<br><br>Piroxantrone – 150 mg/m^2 piroxantrone by intravenous infusion over 1 hour.<br><br>Taxol – 250 mg/m^2 taxol by a 24-hour intravenous infusion.	21-day cycle	Merba	35 (26M, 9F)	62 (42 – 80)	N/A	N/A
				Piro	44 (27M, 17F)	61 (31 – 85)	N/A	N/A
				Taxol	24 (17M, 7F)	61 (38 – 82)	N/A	N/A
Kubota K, 2017 (Kubota et al., 2017)	Carboplatin, Motesanib, Paclitaxel	Once-daily oral motesanib 125 mg or matching placebo; all patients also received paclitaxel 200 mg/m^2 IV and carboplatin area under the concentration-time curve 6 mg/mL·min IV on day 1 of each cycle.	21-day cycle, for ≤6 cycles	Car-Pac-Placebo	204 (147M, 57F)	64 (upper quartile: 58; lower quartile: 69)	Stage IV: 194<br>Recurrent: 10	Former: 108<br>Current: 32<br>Never used: 64
				Car-Pac-Mote	197 (141M, 56F)	65 (upper quartile: 59; lower quartile: 70)	Stage IV: 171<br>Recurrent: 26	Former: 131<br>Current: 17<br>Never used: 49
Zinner RG, 2015 (Zinner et al., 2015)	Bevacizumab, Carboplatin,	Planned chemotherapy doses were pemetrexed 500 mg/m^2; carboplatin, area under the curve = 6, (as of	21-day cycle, after 4 cycles of induction therapy,	Car-Pem	182 (105M, 77F)	65.8 (38.4 – 84.1)	Stage IV: 181<br>Missing: 1	Former/Current: 164<br>Never: 18

	Paclitaxel<br>Pemetrexed	December 31, 2010, maximum possible dose of 900 mg), paclitaxel 200 mg/m^2; bevacizumab<br>15 mg/kg.	maintenance continued<br>until progressive disease,<br>unacceptable toxicity, death, or a desire to withdraw.	Car-Bev-Pac	179 (104M, 75F)	65.4<br>(41.2 – 86.2)	Stage IV: 179	Former/Current: 172<br>Never: 7
**Reference (Publication Year)**	**Drug Combination**	**Delivery / Schedule**	**Treatment Period**	**Dose**	**Number Randomised to Group (M/F)**	**Age, Median (Range)**	**NSCLC Stage**	**Smoker Status (Former/Current/Never)**
Heigener DF, 2013 (Heigener et al., 2013)	Chemotherapy, Sagopilone	Arm A: 3h infusion of 16 mg/m^2 sagopilone<br><br>Arm B: 0.5h infusion of 22 mg/m^2 sagopilone<br><br>Arm C: 3 h infusion of 22 mg/m^2 sagopilone	21-day cycle,<br>for 2 – 6 cycles	S-16,3h	44 (27M, 17F)	63	TNM<br>Stage I: 2<br>Stage II: 0<br>Stage III: 19<br>Stage IV: 23	N/A
				S-22, 0.5h	41 (27M, 14F)	62	TNM<br>Stage I: 1<br>Stage II: 3<br>Stage III: 13<br>Stage IV: 24	N/A
				S-22,3h	43 (29M, 14F)	62	TNM<br>Stage I: 0<br>Stage II: 4<br>Stage III: 15<br>Stage IV: 24	N/A
Wang XJ, 2018 (Jie Wang et al., 2018)	Cisplatin, Endostar, Pemetrexed	Patients in the control group were treated with pemetrexed 500 mg/m^2 IV day 1 and intracavitary cisplatin with a total dose of 75 mg/m^2, days 2, 5 and 8.<br><br>Patients in the treatment group were treated with<br>pemetrexed 500 mg/m^2 IV day 1, intracavitary cisplatin with a total dose of 75 mg/m^2, days 2, 5 and 8, and intracavitary endostar 45mg, days 1, 4 and 7.	21-day cycle,<br>for 3 cycles	Cis-Pem	62 (47M, 15F)	No mean / median<br>(38 – 76)	N/A	N/A
				Cis-Pem-Endostar	66 (49M, 17F)	No mean / median<br>(36 – 75)	N/A	N/A
Eli Lilly and Company, 2019 (Eli	Gefitinib, Pemetrexed	Gef: 250 mg gefitinib taken orally once daily (QD)	21-day cycle, until progressive disease, unacceptable	Gef	65 (24M, 41F)	60.94<br>(S.D.: 9.45)	N/A	N/A
				Gef-Pem	126 (44M, 82F)	62.11		

Lilly and Company, 2019a)		Gef-Pem: 250 mg gefitinib taken orally once daily (QD) and 500 milligrams per square meter (mg/m²) pemetrexed taken intravenously (IV) once every 3 weeks concurrently with Gefitinib QD.	toxicity, death, or a desire to withdraw.			(S.D.: 9.36)		
**Reference (Publication Year)**	**Drug Combination**	**Delivery / Schedule**	**Treatment Period**	**Dose**	**Number Randomised to Group (M/F)**	**Age, Median (Range)**	**NSCLC Stage**	**Smoker Status (Former/Current/Never)**
Douillard JY, 2004 (Douillard, 2004)	BMS-275291, Carboplatin, Paclitaxel	BMS-275291 or placebo was initiated on the same day as paclitaxel/carboplatin and administered on an outpatient basis at a daily oral dosage of 1200 mg (two 600 mg tablets).<br><br>Paclitaxel 200mg/m^2 was administered as a continuous in fusion over 3 h followed by carboplatin calculated using the Calvert formula for a target AUC of 6 mg/(ml min) and given intravenously as a 30 min infusion.	21-day cycle, for ≤8 cycles, until progressive disease, unacceptable toxicity, death, or a desire to withdraw.	Car-Pac-Placebo	37 (26M, 11F)	59.5	Stage IIIB: 3<br>Stage IV: 34	N/A
				Car-Pac-BMS275291	38 (30M, 8F)	63.2	Stage IIIB: 4<br>Stage IV: 34	N/A
Butts CA, 2007 (Butts et al., 2007)	Carboplatin, Cisplatin, Cetuximab, Gemcitabine	Arm A: cetuximab 400mg/m^2 IV over 2 hours at week 1 followed by 250 mg/m^2 weekly (IV over 60 minutes), plus either gemcitabine 1,250 mg/m^2 days 1 and 8 and cisplatin 75 mg/m^2 day 1 every 3 weeks, or gemcitabine 1,000 mg/m^2 (days 1 and 8) and carboplatin area under the concentration-versus-time curve (AUC) of 5 on day 1 of each cycle.<br><br>Arm B: platinum plus gemcitabine identical to arm A, but without cetuximab.	21-day cycle, for ≤6 cycles, until progressive disease, unacceptable toxicity, death, or a desire to withdraw.	Car-Cis-Gem	66 (33M, 33F)	64<br>(35 – 84)	Stage IIIB: 8<br>Stage IV: 55<br>Recurrent: 3	Former/Current: 57<br>Never: 9
				Car-Cis-Gem-Cet	65 (25M, 40F)	66<br>(36 – 84)	Stage IIIB: 5<br>Stage IV: 55<br>Recurrent: 5	Former/Current: 55<br>Never: 10
Fukuda M, 2019 (Fukuda et al., 2019)	Bevacizumab, Chemotherapy, Pemetrexed	All patients received chemotherapy and with either (1) pemetrexed (500 mg/m^2; a 10-min intravenous infusion; every 21 days) or (2)	21-day cycle, until progressive disease, unacceptable	CT-Pem	20 (12M, 8F)	77.5<br>(75 – 82)	Stage IIIB: 0<br>Stage IV: 15<br>Postoperative recurrence: 5	Former/Current: 12<br>Never: 8

Reference (Publication Year)	Drug Combination	Delivery / Schedule	Treatment Period	Dose	Number Randomised to Group (M/F)	Age, Median (Range)	NSCLC Stage	Smoker Status (Former/Current/Never)
		pemetrexed plus bevacizumab (15 mg/kg; an intravenous infusion; every 21 days)	toxicity, death, or a desire to withdraw.	CT-Pem-Bev	20 (11M, 9F)	78.5 (75 – 83)	Stage IIIB: 1 Stage IV: 15 Postoperative recurrence: 4	Former/Current: 10 Never: 10
Passardi A, 2008 (Passardi et al., 2008)	Docetaxel, Gemcitabine	The experimental regimen (arm A), defined in the phase I trial as docetaxel 70 mg/m^2 on day 1 and gemcitabine 900 mg/m^2 on days 3–8, or the empirical regimen (arm B), consisting of gemcitabine 900 mg/m^2 on days 1 and 8, and docetaxel 70 mg/m^2 on day 8.	21-day cycle, for ≤8 cycles, until progressive disease, unacceptable toxicity, death, or a desire to withdraw.	Gem3,8-Doc1	40 (33M, 7F)	63 (35 – 77)	N/A	N/A
				Gem1,8-Doc8	41 (32M, 9F)	63 (48 – 74)	N/A	N/A
Gatzemeier U, 2004 (Gatzemeier et al., 2004)	Cisplatin, Gemcitabine, Trastuzumab	All patients received gemcitabine 1250 mg/m^2 IV over 30 min on days 1 and 8 of a 21-day cycle in combination with cisplatin 75 mg/m^2 IV over 1 h on day 1 with mannitol diuresis.<br><br>In the experimental arm, trastuzumab was administered before CT as a 4 mg/kg initial dose IV over 90 min on day 1, followed by 2 mg/kg IV over 30 min weekly. After completion of CT, Trastuzumab was administered alone.	21-day cycle, CT for ≤6 cycles, Trastuzumab until disease progression	Cis-Gem	50 (30M, 20F)	60 (35 – 76)	Stage IB: 0 Stage IIIB: 6 With effusion: 5 Stage IV: 39	N/A
				Cis-Gem-Tras	51 (33M, 18F)	57 (35 – 76)	Stage IB: 1 Stage IIIB: 9 With effusion: 3 Stage IV: 38	N/A
Park C, 2017 (Park et al., 2017)	Cisplatin, Docetaxel, Pemetrexed	Patients with chemotherapy-naive NSq-NSCLC were randomized into 2 groups: cisplatin 70 mg/m^2 combined with either docetaxel 60 mg/m^2 (Doc-Cis group) or pemetrexed 500 mg/m^2 (Pem-Cis group).	21-day cycle, for ≤4 cycles, until progressive disease, unacceptable toxicity, death, or a desire to withdraw.	Cis-Doc	71 (50M, 21F)	63.6 (S.D. = 9.7)	Stage IIIB: 3 Stage IV: 68	Former/Current: 35 Never: 14 Missing: 22
				Cis-Pem	77 (53M, 24F)	63.0 (S.D. = 8.9)	Stage IIIB: 5 Stage IV: 72	Former/Current: Never: 37 Missing: 26
Movsas B, 2005 (Movsas et al., 2005)	Amifostine, Carboplatin, Paclitaxel	Paclitaxel 225 mg/m^2 was administered intravenously (IV; 3-hour infusion) followed by carboplatin (AUC 6) on days 1 and 22.	21-day cycle, for 2 cycles (CT)	Car-Pac	122 (79M, 43F)	<60: 51 60-64: 24 64-69: 25 ≥70: 22	Stage IIA: 1 Stage IIB: 1 Stage IIIA: 59 Stage IIIB: 61	N/A

		This was followed by concurrent weekly paclitaxel (50 mg/m^2 IV during 1 hour) and carboplatin (AUC 2) with hyperfractionated RT starting on day 43.<br><br>In the AM arm, AM 500 mg IV during 5 minutes was administered before the afternoon treatment 4 days per week (Monday to Thursday).		Car-Pac-Ami	120 (71M, 49F)	<60: 45<br>60 – 64: 32<br>64 – 69: 15<br>≥70: 28	Stage IIA: 1<br>Stage IIB: 1<br>Stage IIIA: 61<br>Stage IIIB: 57	N/A
**Reference (Publication Year)**	**Drug Combination**	**Delivery / Schedule**	**Treatment Period**	**Dose**	**Number Randomised to Group (M/F)**	**Age, Median (Range)**	**NSCLC Stage**	**Smoker Status (Former/Current/Never)**
Jänne P, 2014 (Jänne et al., 2014)	Cisplatin, Gemcitabine, LY293111	Patients were randomly assigned to receive<br>(1) LY293111 200 mg twice daily,<br>(2) LY293111 600 mg twice daily or<br>(3) placebo,<br>followed by gemcitabine (1250 mg/m^2; days 1 and 8), and cisplatin (75 mg/m^2, day 1), every 21 days.	21-day cycle, for ≤6 cycles,<br>until progressive disease, unacceptable toxicity, death, or a desire to withdraw.	Cis-Gem-Placebo	61 (37M, 24F)	60.9<br>(27.8 – 76.6)	Stage IIIB: 7<br>Stage IV: 54	N/A
				Cis-Gem-200LY	70 (42M, 28F)	62.4<br>(36.7 – 81.2)	Stage IIIB: 14<br>Stage IV: 56	N/A
				Cis-Gem-600LY	64 (48M, 16F)	60.7<br>(28.5 – 87.8)	Stage IIIB: 9<br>Stage IV: 55	N/A
Groen HJ, 2011 (Groen et al., 2011)	Carboplatin, Celecoxib, Docetaxel	All patients received carboplatin area under the curve (AUC) of 6mg/mL/min intravenously and docetaxel 75 mg//m^2 both on day 1 of each 21-day cycle, repeated for five cycles.<br><br>Patients were randomly assigned by telephone to receive celecoxib 400 mg orally twice per day or placebo. Celecoxib started on day 1 of the first chemotherapy cycle.	21-day cycle, for 5 cycles, celecoxib continued until progression of disease or a maximum of 3 years.	Car-Doc	280 (171M, 109F)	61<br>(33 – 84)	Stage IIIB: 44<br>Stage IV: 236	N/A
				Car-Doc-Celeco	281 (184M, 97F)	62<br>(40 – 84)	Stage IIIB: 49<br>Stage IV: 232	N/A
Currow D, 2017 (Currow et al., 2017)	Anamorelin, Placebo	Patients enrolled in ROMANA 1 or ROMANA 2 were randomized (2 : 1) to 12 weeks of daily oral anamorelin 100 mg or placebo. Patients enrolled in ROMANA 3 continued to receive treatment once daily for an additional 12 weeks.	Until progressive disease, unacceptable toxicity, death, or a desire to withdraw.	Anamorelin	345<br>(262M, 83F)	62.0<br>(S.D. = 8.5)	Stage IIIA: 34<br>Stage IIIB: 66<br>Stage IV: 244<br>Missing: 1	N/A
			Until progressive disease, unacceptable	Placebo	168<br>(125M, 43F)	62.2<br>(S.D. = 8.4)	Stage IIIA: 18<br>Stage IIIB: 35<br>Stage IV: 115	N/A

			toxicity, death, or a desire to withdraw.				Missing: 0	
**Reference (Publication Year)**	**Drug Combination**	**Delivery / Schedule**	**Treatment Period**	**Dose**	**Number Randomised to Group (M/F)**	**Age, Median (Range)**	**NSCLC Stage**	**Smoker Status (Former/Current/Never)**
Langer CJ, 2017 (Langer et al., 2017)	Cisplatin, Pemetrexed, Placebo	All patients first entered the induction stage which they received 500 mg/m^2 pemetrexed IV and 75 mg/m^2 cisplatin IV, on Day 1 of each 21-day cycle for 4 cycles.<br><br>Then they either received 500 mg/m^2 pemetrexed IV or placebo (normal saline) IV, plus best supportive care, as maintenance.	21-day cycle, for ≤ 4 cycles until progression of disease unacceptable toxicity, death, or a desire to withdraw.	Cis-Pem-Pem	Only summarised as overall<br><br>939 (577M, 363F)	Only summarised as overall<br><br>61.3 (24.4 – 83.0)	N/A	Only summarised as overall<br><br>Former: 757<br>Never used: 175<br>Unknown: 7
				Cis-Pem-Pla			N/A	
Kotsakis A, 2015 (Kotsakis et al., 2015)	Bevacizumab, Cisplatin, Docetaxel, Gemcitabine, Vinorelbine	Arm A: 3 cycles of VCB (vinorelbine 60 mg/m^2 day 1 and 8, cisplatin 80 mg/m^2 on day 1 and bevacizumab 15 mg/kg on day 1), followed by 3 cycles of DGB (docetaxel 75 mg/m^2 on day 1, gemcitabine 1100 mg/m^2 on day 1 and 8 and bevacizumab 15 mg/kg on day 1)<br><br>Arm B: 6 cycles of DCB (docetaxel 75 mg/m^2, cisplatin 80 mg/m^2 and bevacizumab 15 mg/kg all on day 1).	21-day cycle, for 6 cycles	VCB -> DGB	38 (29M, 9F)	60.0 (36 – 77)	Stage IIIB (with pleural effusion): 9<br>Stage IV: 29	Former: 12<br>Current: 18<br>Never used: 8
				DCB	39 (28M, 11F)	58.0 (39 – 75)	Stage IIIB (with pleural effusion): 6<br>Stage IV: 33	Former: 11<br>Current: 21<br>Never used: 7
Eli Lilly and Company, 2015 (Eli Lilly and Company, 2015)	Carboplatin, Cetuximab, Taxane (Paclitaxel/Docetaxel)	TC Arm: Taxane was paclitaxel 225 mg/m^2 infused over 180 minutes on Day 1 and subsequently every 3 weeks or docetaxel 75 mg/m^2 infused over 60 minutes on Day 1 and subsequently every 3 weeks. Carboplatin was infused over 30 minutes on Day 1 and subsequently every 3 weeks.<br><br>TC-Cel Arm: Cetuximab was administered at an initial dose (Week 1) of 400 mg/m^2 IV infusion (infused over 120 minutes) and a weekly maintenance dose of 250 mg/m^2 IV	21-day cycle, for ≤ 6 cycles until progression of disease unacceptable toxicity, death, or a desire to withdraw.	Tax-Car	338 (204M, 134F)	63.9 (S.D.: 10.3)	N/A	N/A
				Tax-Car-Cel	338 (192M, 146F)	64.0 (S.D.: 10.0)	N/A	N/A

		infusion (infused over 60 minutes). Taxane and carboplatin were given as described above.						
**Reference (Publication Year)**	**Drug Combination**	**Delivery / Schedule**	**Treatment Period**	**Dose**	**Number Randomised to Group (M/F)**	**Age, Median (Range)**	**NSCLC Stage**	**Smoker Status (Former/Current/Never)**
GSK, 2019 (GlaxoSmith Kline, 2019)	Carboplatin, Cisplatin, Docetaxel, GSK3052230, Paclitaxel, Pemetrexed	**Arm A: GSK3052230, paclitaxel/carboplatin** Subject received 5/10/20 mg/kg GSK3052230 as 30-minute intravenous (IV) infusion once a week (Day 1, Day 8, Day 15) of each 21-day cycle and 200 mg/m^2 paclitaxel (constant infusion for 3 hrs) and 900 mg carboplatin (constant infusion for 30 to 60 minutes) IV on Day 1 of each 21-day cycle.<br><br>**Arm B: GSK3052230, docetaxel** Subject received 5/10/20 mg/kg GSK3052230 as 30-minute intravenous (IV) infusion once a week (Day 1, Day 8, Day 15) of each 21-day cycle and 75 mg/m^2 docetaxel as 1 hour IV infusion on Day 1 of each 21-day cycle.<br><br>**Arm C: GSK3052230, pemetrexed and cisplatin** Subject received 10/15/20 mg/kg GSK3052230 as 30 minute IV infusion once a week (Day 1, Day 8, Day 15) of each 21 day cycle, 500 mg/m^2 pemetrexed IV infusion over 10 minutes on Day 1 of each 21 day cycle followed 30 minutes later by 75 mg/m^2 cisplatin infused over 2 hours.<br><br>*GSK3052230 A clear to opalescent, colorless to pale yellow solution for IV infusion once	21-day cycle, For Arm A, paclitaxel/carboplatin it was limited to 4 to 6 cycles, others until progression of disease unacceptable toxicity, death, or a desire to withdraw.	5GSK-Car-Pac	3 (3M)	71.0 (S.D.: 5.29)	N/A	N/A
				10GSK-Car-Pac	3 (3M)	71.7 (S.D.: 5.03)	N/A	N/A
				20GSK-Car-Pac	14 (13M, 1F)	65.0 (S.D.: 7.55)	N/A	N/A
				5GSK-Doc	3 (3M)	58.0 (S.D.: 8.19)	N/A	N/A
				10GSK-Doc	3 (3M)	67.0 (S.D.: 12.00)	N/A	N/A
				20GSK-Doc	3 (2M, 1F)	65.7 (S.D.: 5.77)	N/A	N/A
				10GSK-Cis-Pem	3 (2M, 1F)	72.3 (S.D.: 7.51)	N/A	N/A
				15GSK-Cis-Pem	25 (17M, 8F)	57.1 (S.D.: 13.9)	N/A	N/A

Reference (Publication Year)	Drug Combination	Delivery / Schedule	Treatment Period	Dose	Number Randomised to Group (M/F)	Age, Median (Range)	NSCLC Stage	Smoker Status (Former/Current/Never)
		weekly (Day 1, Day 8, Day 15) in each 21-day cycle with unit dose strengths/dose level of 5, 10, 15, and 20 mg/kg supplied in a sterile 25 mL glass vial.						
				20GSK-Cis-Pem	8 (6M, 2F)	70.9 (S.D.: 6.06)	N/A	N/A
Lara PN, 2016 (Lara et al., 2016)	Carboplatin, Erlotinib, Paclitaxel	Arm 1: 150 mg of erlotinib orally daily	21-day cycle, for 4 cycles, erlotinib continued until progression of disease unacceptable toxicity, death, or a desire to withdraw.	Erlo	33 (14M, 19F)	74.9 (45.2 – 84.9)	Stage IIIB: 1<br>Stage IV: 32	Former: 17<br>Current: 10<br>Never used: 6
		Arm 2: 150 mg of erlotinib orally daily on days 2 – 16 plus four cycles of carboplatin (area under the curve = 5 on day 1) and paclitaxel (200 mg/m^2 IV on day 1) followed by 150 mg of erlotinib orally.		Erlo-Car-Pac	26 (10M, 16F)	70.8 (40.9 – 85.9)	Stage IIIB: 1<br>Stage IV: 25	Former: 11<br>Current: 9<br>Never used: 6
Wu YL, 2020 (Wu et al., 2020)	Carboplatin, Cisplatin, Gefitinib, Pemetrexed, Tepotinib,	Phase 1b: Tepotinib 300 or 500 mg and Gefitinib 250 mg tablets orally once daily over a 21-day cycle<br><br>Phase 2 with MET + T790 Negative: (i) Tepotinib 500 mg and Gefitinib 250 mg orally once daily over a 21-day cycle<br>or (ii) 500 mg/m^2 pemetrexed IV over 10 minutes in combination with cisplatin (75 mg/m^2 as an intravenous infusion over 2 hours) or carboplatin IV at a dose of AUC 5 or AUC 6 on Day 1<br><br>Phase 2 with MET + T790 Positive: Tepotinib 500 mg and Gefitinib 250 mg tablets orally once daily over a 21-day cycle	21-day cycle, for 6 cycles (4 cycles if followed by pemetrexed as maintenance), erlotinib continued until progression of disease unacceptable toxicity, death, or a desire to withdraw.	1b-300Tep-Gef	6 (3M, 3F)	N/A	N/A	N/A
				1b-500Tep-Gef	12 (5M, 7F)	N/A	N/A	N/A
				2Neg-Tep-Gef	31 (11M, 20F)	N/A	N/A	N/A
				2Neg-Pem-Car/Cis	24 (12M, 12F)	N/A	N/A	N/A
				2Pos-Tep-Gef	15 (5M, 10F)	N/A	N/A	N/A
Umsawasdi T, 1989)	Cisplatin, Cyclophospha	Pateints were randomised to receive doxorubicin either as a 6-hour	21-day cycle	Weekly-Dox	51 (34M, 17F)	59 (41 – 77)	N/A	N/A
				Triweekly-Dox	51 (37M, 14F)	54 (33 – 78)	N/A	N/A

(Umsawasdi et al., 1989)	mide, Doxorubicin	continuous infusion every 3 weeks or as a 30-minute infusion every week. All patients received doxorubicin on day 1 followed by cyclophosphamide and cisplatin on day 2.						
**Reference (Publication Year)**	**Drug Combination**	**Delivery / Schedule**	**Treatment Period**	**Dose**	**Number Randomised to Group (M/F)**	**Age, Median (Range)**	**NSCLC Stage**	**Smoker Status (Former/Current/Never)**
Cortot AB, 2020 (Cortot et al., 2020)	Bevacizumab, Docetaxel, Paclitaxel	Within 28 days after selection, patients were randomised to receive either 90 mg/m^2 of paclitaxel (D1, D8, D15) and 10 mg/kg of bevacizumab (D1, D15) every four weeks or 75 mg/m^2 of docetaxel every three weeks.	Until progression of disease unacceptable toxicity, death, or a desire to withdraw.	Bev-Pac	111 (78M, 33F)<br><br>Prior exposure to Bev: 34	59.6<br>(18.6 – 81.8)	N/A	Former/Current: 102<br>Never: 9
				Doc	55 (42M, 13F)<br><br>Prior exposure to Bev: 17	59.7<br>(35.8 – 78.9)	N/A	Former/Current: 46<br>Never: 9
AstraZeneca, 2021 (AstraZeneca, 2021)	Docetaxel, Selumetinib	Three placebo / 25mg selumetinib capsules were administered orally uninterrupted twice daily in combination with docetaxel 75 mg/m^2 intravenously administered on day 1 of each 21-day cycle.	21-day cycle, until progression of disease unacceptable toxicity, death, or a desire to withdraw.	Doc-Plac	256 (145M, 111F)	60.9<br>(S.D.: 8.08)	N/A	N/A
				Doc-Selu	254 (158M, 96F)	61.9<br>(S.D.: 8.48)	N/A	N/A
Johnson DH, 2004 (Johnson et al., 2004)	Bevacizumab, Carboplatin, Paclitaxel	Control: carboplatin ad paclitaxel alone. Paclitaxel (200mg/m^2 was administered over 3 hours every 3 weeks. Carboplatin dosing was based on the Calvert formula with a target area under the curve of 6 mg/mL X min.<br><br>Low Dose: Carboplatin and paclitaxel plus low-dose (7.5 mg/kg) bevacizumab.<br><br>High Dose: Carboplatin and paclitaxel plus high-dose (15 mg/kg) bevacizumab.	≤ six cycles of carboplatin and paclitaxel.<br><br>Patients were followed for survival information every 2 months until death or loss to follow-up.	Car-Pac	32 (24M, 8F)	N/A	Stage IIIB: 6<br>Stage IV: 26	N/A
				Car-Pac-7.5Bev	32 (20M, 12F)	N/A	Stage IIIB: 2<br>Stage IV: 30	N/A
				Car-Pac-15Bev	35 (16M, 19F)	N/A	Stage IIIB: 7<br>Stage IV: 28	N/A
Eli Lilly and Company, 2022 (Eli	Cisplatin, Gemcitabine, Necitumumab	Cisplatin: 75 mg/m^2 IV on Day 1 of every 3-week cycle.	21-day cycle, cis/gem for ≤6 cycles,	Cis-Gem	548 (458M, 90F)	62.0<br>(32 – 86)	Stage IIIB: 1<br>Stage IV: 546<br>Missing: 1	Former: 26<br>Current: 495

Lilly and Company, 2022)		Gemcitabine: 1250 mg/m^2 on Days 1 and 8 of every 3-week cycle.<br><br>Necitumumab: 800 mg I.V. infusion on Days 1 and 8 of every 3-week cycle.	necitumumab until progression of disease unacceptable toxicity, death, or a desire to withdraw.					Never used: 27<br>Missing: 0
				Cis-Gem-Nec	545<br>(450M, 95F)	62.0<br>(32 – 84)	Stage IIIB: 1<br>Stage IV: 543<br>Missing: 1	Former: 18<br>Current: 500<br>Never used: 26<br>Missing: 1
**Reference (Publication Year)**	**Drug Combination**	**Delivery / Schedule**	**Treatment Period**	**Dose**	**Number Randomised to Group (M/F)**	**Age, Median (Range)**	**NSCLC Stage**	**Smoker Status (Former/Current/Never)**
Eli Lilly and Company, 2021 (Eli Lilly and Company, 2021)	Cisplatin, Pemetrexed, Necitumumab	Cisplatin: 75 mg/m2 I.V. on Day 1 of every 3-week cycle.<br><br>Pemetrexed: 500 mg/m2 I.V. on Day 1 of every 3-week cycle.<br><br>Necitumumab: 800 mg (absolute dose) on Days 1 and 8 of every 3-week cycle, administered as an I.V. infusion	21-day cycle, cis/pem for ≤6 cycles, necitumumab suntil progression of disease unacceptable toxicity, death, or a desire to withdraw.	Cis-Pem	318 (210M, 108F)	60.0<br>(34 – 88)	Stage IIIB: 1 1<br>Stage IV: 307<br>Missing: 0	Former: 27<br>Current: 238<br>Never used: 53
				Cis-Pem-Nec	315 (214M, 101F)	61.0<br>(26 – 84)	Stage IIIB: 9<br>Stage IV: 305<br>Missing: 1	Former: 26<br>Current: 238<br>Never used: 51
Eli Lilly and Company, 2019 (Eli Lilly and Company, 2019b)	Carboplatin, Paclitaxel, Necitumumab	Carboplatin AUC6 administered IV on Day 1 of every 3-week cycle.<br><br>Paclitaxel 200 mg/m² administered IV on Day 1 of every 3-week cycle.<br><br>Necitumumab 800 mg administered intravenously (IV) on Days 1 and 8 of every 3-week cycle.	21-day cycle, car/pac for ≤6 cycles, necitumumab until progression of disease unacceptable toxicity, death, or a desire to withdraw.	Car-Pac	57 (44M, 13F)	64.7<br>(S.D.: 8.27)	N/A	N/A
				Car-Pac-Nec	110 (87M, 23F)	65.5<br>(S.D.: 9.36)	N/A	N/A
